# Blocking CCN2 Reduces Progression of Sensorimotor Declines and Fibrosis in a Rat Model of Chronic Repetitive Overuse

**DOI:** 10.1002/jor.24337

**Published:** 2019-06-20

**Authors:** Mary F. Barbe, Brendan A. Hilliard, Sean P. Delany, Victoria J. Iannarone, Michele Y. Harris, Mamta Amin, Geneva E. Cruz, Yeidaliz Barreto‐Cruz, Ngih Tran, Emily P. Day, Lucas J. Hobson, Soroush Assari, Steven N. Popoff

**Affiliations:** ^1^ Department of Anatomy and Cell Biology, Lewis Katz School of Medicine Temple University Philadelphia Pennsylvania 19140; ^2^ Department of Mechanical Engineering, College of Engineering Temple University Philadelphia Pennsylvania 19122

**Keywords:** work‐related musculoskeletal disorders (WMSDs), tendinopathy, nerve, muscle, tendon

## Abstract

Fibrosis may be a key factor in sensorimotor dysfunction in patients with chronic overuse‐induced musculoskeletal disorders. Using a clinically relevant rodent model, in which performance of a high demand handle‐pulling task induces tissue fibrosis and sensorimotor declines, we pharmacologically blocked cellular communication network factor 2 (CCN2; connective tissue growth factor) with the goal of reducing the progression of these changes. Young adult, female Sprague–Dawley rats were shaped to learn to pull at high force levels (10 min/day, 5 weeks), before performing a high repetition high force (HRHF) task for 3 weeks (2 h/day, 3 days/week). HRHF rats were untreated, or treated in task weeks 2 and 3 with a monoclonal antibody that blocks CCN2 (FG‐3019), or a control immunoglobulin G (IgG). Control rats were untreated or received FG‐3019, IgG, or vehicle (saline) injections. Mean task reach rate and grasp force were higher in 3‐week HRHF + FG‐3019 rats, compared with untreated HRHF rats. Grip strength declined while forepaw mechanical sensitivity increased in untreated HRHF rats, compared with controls; changes improved by FG‐3019 treatment. The HRHF task increased collagen in multiple tissues (flexor digitorum muscles, nerves, and forepaw dermis), which was reduced with FG‐3019 treatment. FG‐3019 treatment also reduced HRHF‐induced increases in CCN2 and transforming growth factor β in muscles. In tendons, FG‐3019 reduced HRHF‐induced increases in CCN2, epitendon thickening, and cell proliferation. Our findings indicate that CCN2 is critical to the progression of chronic overuse‐induced multi‐tissue fibrosis and functional declines. FG‐3019 treatment may be a novel therapeutic strategy for overuse‐induced musculoskeletal disorders. © 2019 The Authors. *Journal of Orthopaedic Research*® published by Wiley Periodicals, Inc. on behalf of Orthopaedic Research Society. J Orthop Res 37:2004–2018, 2019

Chronic overuse‐induced musculoskeletal disorders are also known as work‐related musculoskeletal disorders, repetitive motion disorders, and repetitive strain injuries. They include diagnoses of carpal tunnel syndrome, tendinopathies, and myalgias.^1^ Risk factors include chronic performance of high repetition and high force activities.^2^ There remains a call for effective treatments for these often debilitating disorders.^3^

Cellular communication network factor 2 (CCN2; CTGF) is a secreted matricellular protein with four modular domains that independently interact with different molecules, such as collagen and proteoglycans in the extracellular matrix.^4^ CCN1 (Cyr6) and CCN3 (NOV) are two family members that can have similar or opposing functions in fibrogenic processes as CCN2, respectively.^5^, ^6^, ^7^ Since fibrosis is thought to distort dynamic properties of tissue due to adherence of adjacent structures, it may also contribute to functional and biomechanical tissue declines.^8^, ^9^ Downregulation of CCN2 by anti‐sense or small interfering RNA treatment reduces liver fibrosis and limits hypertrophic scarring without affecting wound healing.^10^, ^11^ Mdx mice (an animal model of Duchenne muscular dystrophy) with a hemizygous CCN2 deletion, or treated with a neutralizing monoclonal antibody that targets the von Willebrand Factor type C (vWC) domain of CCN2 show reduced muscle fibrosis and improved muscle strength.^12^, ^13^ The CCN2 antibody, known as FG‐3019 or Pamrevlumab (FibroGen Inc., San Francisco, CA), has completed Phase 2 clinical trials for idiopathic pulmonary fibrosis (ClinicalTrials.gov Identifier: NCT01890265^14^), and is currently in Phase 2 trials for locally advanced, unresectable pancreatic cancer (NCT02210559^15^) and Duchenne muscular dystrophy (NCT02606136).

We have a clinically relevant rodent model of chronic overuse in which rats perform an operant reaching, grasping and lever‐pulling task for food reward.^16^ Performance of this task at high repetition high force (HRHF) levels for 3–18 weeks induces progressive musculotendinous and nerve fibrosis and sensorimotor declines,^8^, ^17^, ^18^, ^19^ similar to responses seen in patients with upper extremity overuse‐induced musculoskeletal disorders.^1^ Use of ibuprofen or anti‐tumor necrosis factor ɑ drug reduced tissue inflammation in this model.^17^, ^18^ However, they did not fully ameliorate task‐induced functional declines, or return tissue fibrogenic proteins to control levels in HRHF rats. An initial 5‐week shaping period in which rats learn to pull at high force levels also induces elevated levels of extracellular and fibrogenic proteins (periostin‐like factor, CCN2, and transforming growth factor β1 [TGFβ1]), relative to control rats.^17^, ^19^ Thus, we sought here to determine if a monoclonal antibody that binds human and rodent CCN2 would reduce progression of tissue fibrosis and sensorimotor declines induced early in our operant rat model of overuse‐induced musculoskeletal disorders.

## MATERIALS AND METHODS

Detailed methods, including enzyme‐linked immunosorbent assay (ELISA) and antibody specifics, are in Supplementary Methods.

### Animals

Experiments were approved by the Institutional Animal Care and Use Committee in compliance with NIH guidelines. Studies were conducted on young adult female, Sprague–Dawley rats. Rats were food restricted to motivate participation, yet allowed to gain weight over time (Supplementary Fig. 1). Task rats first underwent a 5‐week shaping period to learn a high force lever‐pulling task, before performing HRHF task for 3 weeks. HRHF rats were untreated or treated with CCN2 antibody (FG‐3019) or control human immunoglobulin G (hIgG). Reach limbs were examined from untreated 23 3‐week HRHF, 10 HRHF + hIgG, and 13 HRHF + FG‐3019 rats. Remaining food‐restricted rats were untreated or treated with FG‐3019, hIgG, or saline (29 food‐restricted control [FRC], 22 FRC + Saline, 5 FRC + hIgG, and 5 FRC + FG‐3019 rats, bilaterally). Muscle and serum tissues were also examined from 17 0‐week HRHF rats (euthanized after shaping).

### HRHF Task

Task rats were shaped for 5 weeks to learn a reaching and lever‐pulling task at high force loads at no specified reach rate (ramping upwards from naïve, 10 min/day, 5 days/week). Rats then performed a HRHF reaching and lever‐pulling task for 3 weeks (48% maximum pulling force, 4 reaches/min, 2 h/day, in 30 min intervals, 3 days/week) for food reward.^16^

### Pharmacological Treatments

Three‐week HRHF rats were untreated, or treated in task weeks 2 and 3 with a human anti‐CCN2 monoclonal antibody (FG‐3019; FibroGen Inc.; 40 mg/kg body weight, i.p.; 2×/week, 2 weeks), or human IgG (hIgG, FibroGen Inc.; 50 µl/injection, i.p.). FRC rats were untreated or treated similarly with FG‐3019, hIgG, or vehicle (100 μl saline, i.p., 2×/week).

### Behavioral Assays

HRHF voluntary reach outcomes were recorded continuously during each session and calculated in task week 3 for 12 HRHF, 10 HRHF + FG‐3019, and 5 HFHF + IgG rats. Reflexive grip strength was assayed using a grip strength meter (1027SR‐D58; Columbus Instruments, Columbus, OH), after onset of food restriction, after shaping (HRHF week 0), and HRHF week 3. Maximum grip strength per trial (5 times/limb) is reported. Forelimb sensitivity to mechanical probing was assayed at end of HRHF task week 3 using monofilaments (Stoelting, IL). Mean number of limb withdrawal responses/10 repeats is reported per monofilament.

### ELISAs

Animals were anesthetized and euthanized before blood collection at 36 hours after their last task session. Blood was centrifuged and serum harvested from: 3‐week HRHF groups (13 HRHF, 9 HRHF + FG‐3019, and 5 HRHF + hIgG), FRC groups (11 FRC, 8 FRC + Saline, 5 FRC + hIgG), and seven 0‐week HRHF rats. Serum was assayed using ELISA for collagen type 1, collagen type 3, CCN2, TGFβ1, and inflammatory cytokines (CCL2, CCL3, CXCL2, CXCL5, CXCL10, IL‐10, and IL‐18). Samples were run in duplicate and data reported as pg/ml serum.

Forelimb tissues were collected for ELISA from reach limbs of 3‐week HRHF rats (10 HRHF, 4 HRHF + FG‐3019, and 5 HRHF + hIgG), one limb per FRC rat (16 FRC, 10 FRC + Saline, 5 FRC + hIgG, and 5 FRC + FG‐3019), and seven 0‐week HRHF rats. Samples were homogenized in phosphate‐buffered saline containing proteinase inhibitors, and centrifuged. Supernatants were assayed for collagen types 1 and 3, CCN2, and TGFβ1, as described for serum. Data (picogram of protein) were normalized to microgram of total protein, determined using bicinchoninic acid (BCA; Thermo Fisher Scientific, Waltham, MA).

### Gelatin Zymography and Western Blot Assays

Aliquots of muscles homogenates (equal amounts of protein) were assayed for matrix metalloproteinase (MMP) 2 and MMP9 activity using gelatin zymography under nonreducing conditions. Results were normalized to mean FRC and FRC + saline results and ratios compared. Aliquots were also assayed using Western blotting for CCN1, CCN2, CCN3, phosphorylated ERK (pERK) and total ERK using 4–12% Tris‐Glycine gels under denaturing conditions. Gels were blotted onto nitrocellulose membranes, blocked in 5% BSA/0.05% Tween‐20, and incubated overnight with specific antibodies per protein. After incubation with appropriate secondary antibodies and imaging using Licor and then ImageJ, normalized bands were compared as described in figure legends.

### Immunohistochemistry and Histochemistry

Rats were euthanized, serum and limbs for ELISA removed, and then intracardial perfusion with buffered 4% paraformaldehyde. Reach limbs were collected from 3‐week HRHF rats (13 HRHF, 9 HRHF + FG‐3019, and 5 HRHF + hIgG), at least one limb per FRC rat (16 FRC, 15 FRC + saline, and 5 FRC + hIgG), and ten 0‐week HRHF rats. A piece of flexor digitorum muscle was removed from mid‐ to proximal forearm for cross‐sectional slicing; remaining muscle–tendon‐nerve mass was sectioned longitudinally (15 μm). Subsets of sections on slides were stained/immunostained with hematoxylin, TUNEL, collagen types 1 and 3, CCN2, PAX7, TGFβ1, anti‐human IgG; or were triple‐labeled with SMA, PDGFR, and tcf4; followed by appropriate secondary antibodies. Forepaws were fixed by immersion, paraffin embedded, sectioned longitudinally (5 μm), placed onto charged slides, and stained with hematoxylin or Masson's Trichrome.

### Histomorphometry

Quantification was performed in 3 fields/tissue by individuals blinded to group assignment, using computerized image analysis systems (Bioquant Corporation, Nashville, TN). In muscles, collagen types 1 and 3, and CCN2 immunostaining were quantified in cross‐sections using a thresholded pixel count. CCN2 was quantified similarly in longitudinal cryosectioned tendons. CD68, PAX7, TGFβ1, and TUNEL^+^ cells, and αSMA^+^/tcf4^+^/PDGFR^+^ cells were counted in muscle cross‐sections. Shortest diameters of myofibers (mean circular mil) were assayed in collagen/DAPI immunostained cross‐sections. Longitudinal sections were used to count CD68^+^ cells within median nerves, epitendon thickness and cellularity, and endotendon cellularity, at wrist level after hematoxylin staining. Cell shape factor analysis (Bioquant Corporation) was used to assay if endotendon cells were more spindle‐shaped (<0.5) versus rounded (>0.05). Percent area with collagen staining around median nerves and in digits’ upper dermis was quantified after Masson's Trichrome staining of paraffin‐embedded and longitudinally cut forepaws.^20^

### Statistical Analyses

A power analysis from past work was first performed and showed a minimum of 4–5/group was needed.^17^, ^20^ All data are expressed as mean ± SEM. *p* < 0.05 was considered significant. Unpaired, two‐tailed *t* tests were used to compare 0‐week HRHF and FRC results; one‐way analysis of variance (ANOVAs) were used to compare operant behavior and Western blot densitometry results; and two‐way ANOVAs were used to analyze grip strength, forepaw mechanical allodynia, ELISA, and histological data. Sidak multiple comparison tests were used for planned post hoc assays (untreated HRHF rats versus untreated FRC rats; HRHF + hIgG to FRC + hIgG; HRHF + FG‐3019 to FRC + FG‐3019 for behavioral or to FRC + saline for tissue data; and HRHF + FG‐3019 to both untreated HRHF and HRHF + hIgG rats). Behavioral results were correlated to collagen staining/levels in tissues using Pearson's or Spearman's *r* tests.

## RESULTS

### Improved Sensorimotor Declines

**Figure 1:**
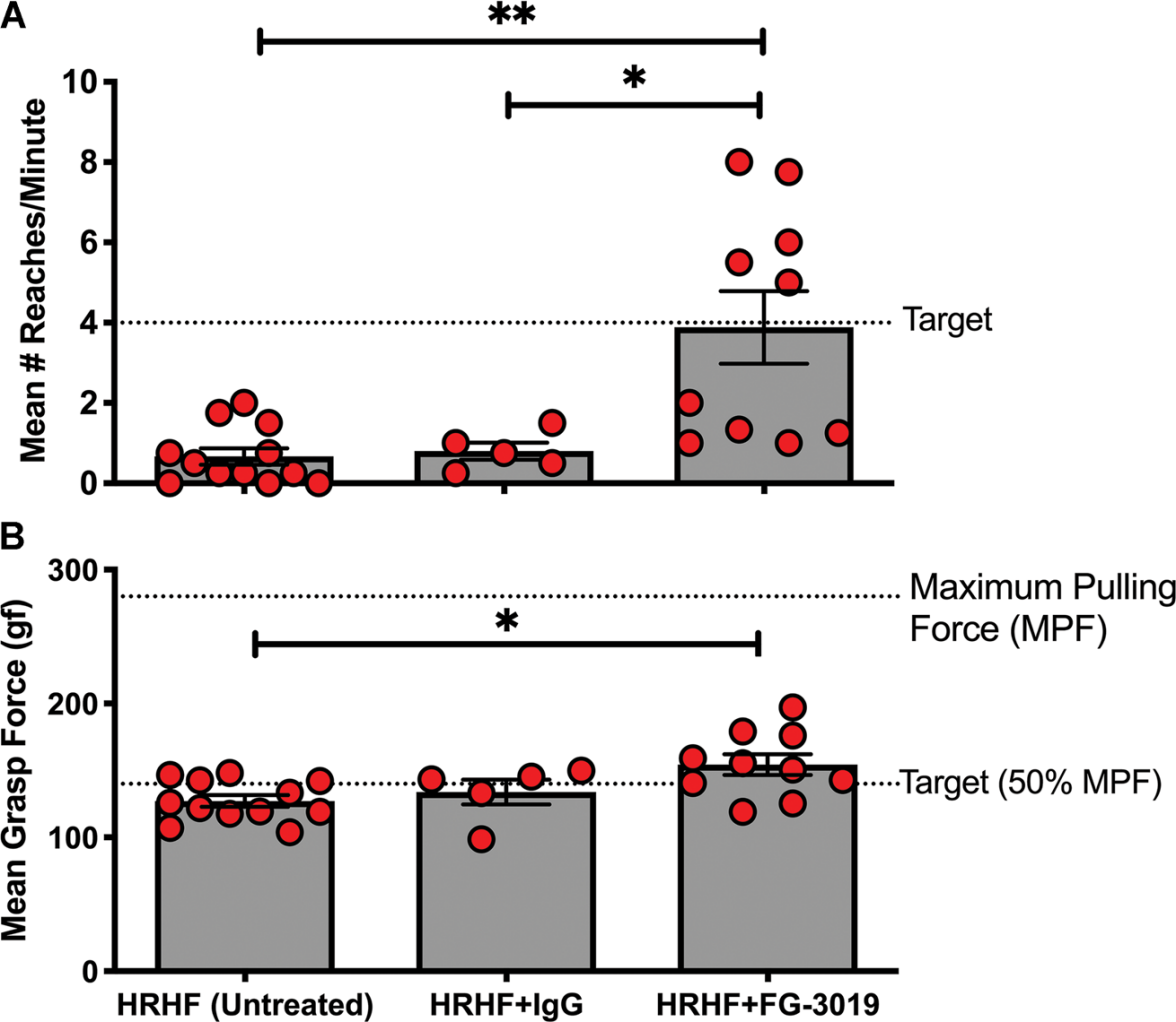
Operant performance behaviors assayed in task week 3 in rats performing a high repetition high force (HRHF) reaching and grasping a lever bar task. (A) Target reach rate was 4 reaches/min. (B) Mean voluntary grasp force on the lever bar. *p < 0.05, and **p < 0.01 compared with groups as shown. FRC, food‐restricted control; IgG, immunoglobulin G [Color figure can be viewed at wileyonlinelibrary.com]

Three‐week HRHF and HRHF + hIgG rats were unable to meet the target reach rate (Fig. 1A). Mean reach rate increased to target levels in HRHF + FG‐3019 rats (Fig. 1A). Mean voluntary grasp force was also higher in HRHF + FG‐3019 rats, compared with untreated HRHF rats (Fig. 1B).

**Figure 2:**
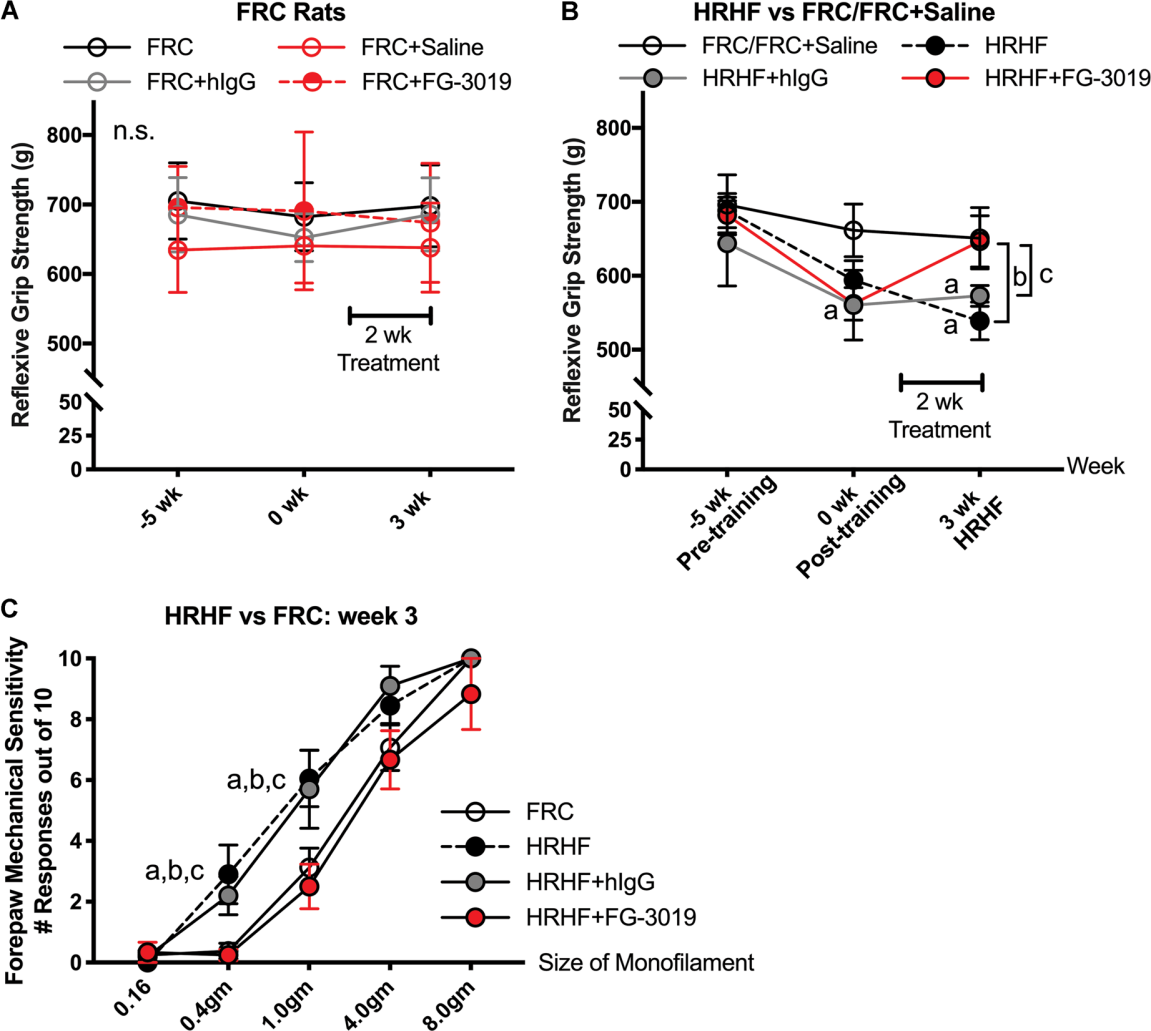
Reflexive grip strength and forepaw mechanical sensitivity. (A) and (B) Reflexive grip strength in food‐restricted control (FRC) and HRHF task groups. HRHF rats were tested at a naïve timepoint immediately prior to the 5‐week shaping period, immediately post‐shaping (task week 0) and after performing the HRHF task for 3 weeks, with subsets receiving treatments in the final 2 weeks. FRC rats were tested at similar times. (C) Forepaw mechanical sensitivity assayed in task week 3 using nylon monofilaments with milliNewton (mN) sizes shown. a: *p* < 0.05, compared with matched FRC group; b: *p* < 0.05, compared with untreated HRHF rats; c: *p* < 0.05, compared with HRHF + hIgG rats; n.s., not significant. Number of limbs tested per group: HRHF, *n* = 12; HRHF + FG‐3019, *n* = 10–12; HRHF + hIgG, *n* = 10; FRC, *n* = 10–25; FRC + Saline, *n* = 10; FRC + hIgG, *n* = 10, and FRC + FG‐3019, *n *= 10, bilateral results included for latter two groups [Color figure can be viewed at wileyonlinelibrary.com]

Reflexive grip strength did not differ between FRC groups (Fig. 2A), yet declined immediately after shaping (week 0) in HRHF rats, compared with baseline and FRCs (Fig. 2B). These declines persisted in 3‐week HRHF and HRHF + hIgG rats, yet improved in HRHF + FG‐3019 rats (Fig. 2B). Forepaw mechanical sensitivity was increased in HRHF rats in response to probing with monofilaments sized 3.92 and 9.81 mN, compared with FRCs (Fig. 2C). This hypersensitivity was not present in HRHF + FG‐3019 rats.

### Reductions in HRHF‐Induced Increases in Muscle Collagen

**Figure 3:**
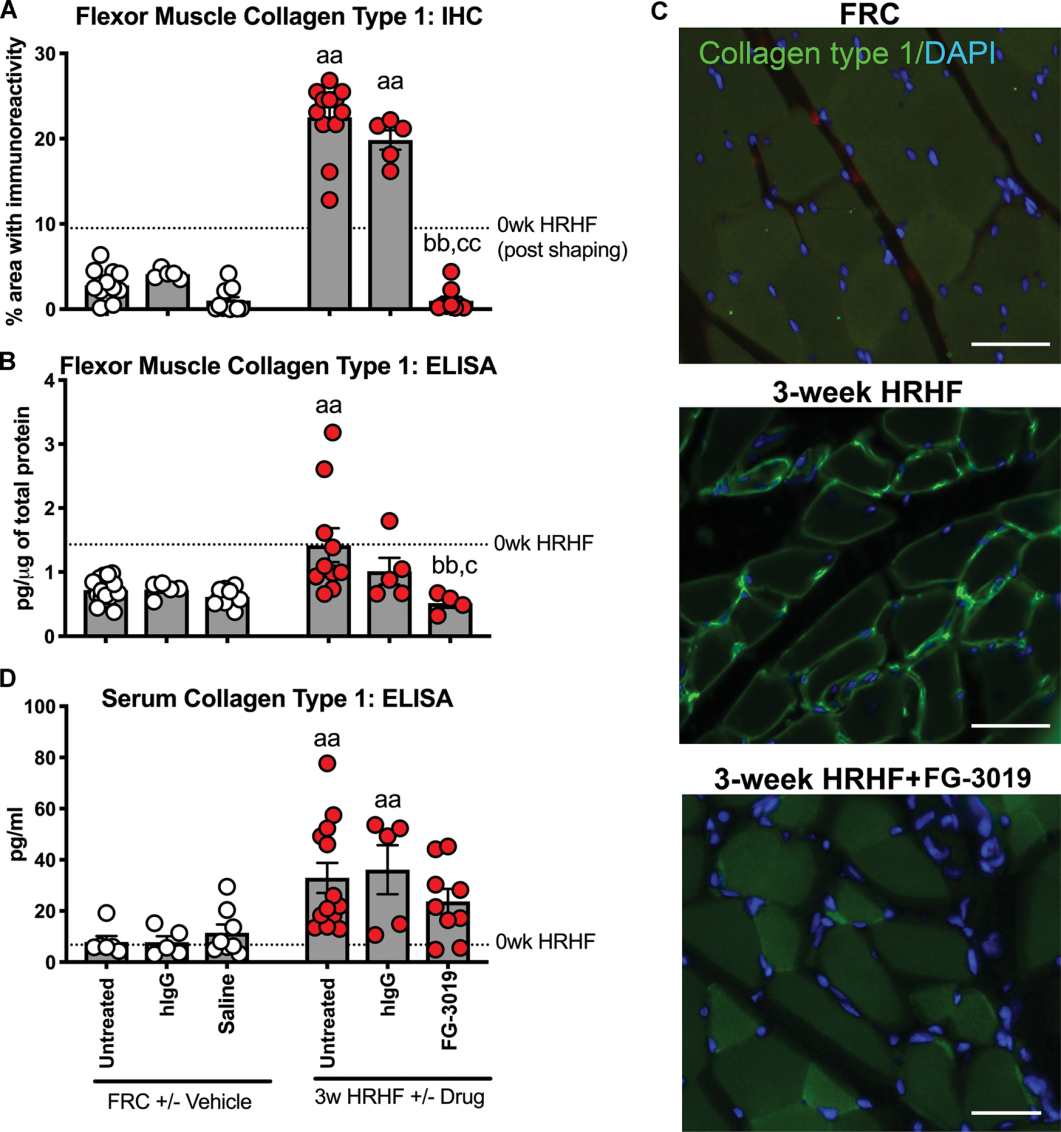
Collagen type 1 in flexor digitorum muscle and serum. (A) Percent area with collagen type 1 immunoreactivity (green) and 4′,6‐diamidino‐2‐phenylindole (DAPI) nuclear stain (blue) in cross‐sectionally cut muscles of food‐restricted control (FRC), 3‐week untreated high repetition high force (HRHF) and HRHF + FG‐3019‐treated rats. (B) Enzyme‐linked immunosorbent assay (ELISA) detected levels in muscles. (C) Collagen type 1 serum levels, tested using ELISA. aa: *p* < 0.01, compared with matched FRC group; bb: *p* < 0.01, compared with HRHF rats; c: *p* < 0.05, and cc: *p* < 0.01, compared with HRHF + hIgG rats. Scale bars = 50 μm. The 0‐week HRHF rat results from Supplementary Table 1 are indicated with dotted lines [Color figure can be viewed at wileyonlinelibrary.com]

We first examined muscles of 0‐week HRHF rats to determine the effects of shaping for 5 weeks to the high force pulling level, and observed increased collagen (immunohistochemically and ELISA assayed) in flexor digitorum muscles, compared with FRCs (*p* < 0.05 each, Supplementary Table 1 and Fig. 3). Collagen type 1 was further increased in muscles of 3‐week HRHF and HRHF + hIgG rats, compared with FRC and FRC + hIgG rats (immunohistochemically and ELISA assayed; Fig. 3A and B). HRHF‐induced increases improved in FG‐3019 rats (Fig. 3A and B). Increased collagen type 1 was localized around myofibers in HRHF rats, relative to other groups (Fig. 3C). Collagen type 1 was detectable in serum, which showed similar responses as muscles (Fig. 3D). Serum and muscle collagen type 3 did not differ between groups (Table 1).

**Table 1:** Additional Fibrogenic Protein and Myofiber Diameter Findings in Serum, and Flexor Digitorum Muscle and Tendons

Analyte and Tissue	FRC Untreated (*n *= 5–9)	FRC + hIgG (*n *= 5)	FRC + Saline (*n *= 4–7)	3‐Week HRHF Untreated (*n *= 8–10)	3‐week HRHF + hIgG (*n *= 5)	3‐Week HRHF + FG‐3019 (*n *= 4–7)
Serum collagen type 3 (pg/ml) (*n *= 5–7/group)*	75.93 ± 9.39	92.96 ± 29.38	74.32 ± 23.99	114.47 ± 18.43	106.90 ± 36.41	137.12 ± 18.71
Muscle collagen type 3, pg/μg total protein* (*n *= 4–9/group)	0.02 ± 0.003	0.03 ± 0.001	0.03 ± 0.005	0.08 ± 0.03	0.05 ± 0.01	0.02 ± 0.004
Muscle myofibroblasts	0.67 ± 0.67	NT	0.17 ± 0.17	**1.25 ± 0.38** ^a^	NT	0.44 ± 0.24
SMA^+^PDGFR^+^tcf4^+^ cells/field of 0.26 mm^2^						
Tendon CCN2, pg/μg total protein* (*n *= 4–10/group)	0.09 ± 0.05	0.19 ± 0.03	0.26 ± 0.06	**0.49 ± 0.17** ^a^	**0.53 ± 0.03** ^a^	**0.20 ± 0.07** ^b^ ^**,**^ ^c^
Endotendon: no. of cells/mm^2^	0.001 ± 0.0006	0.001 ± 0.0003	0.001 ± 0.0003	0.002 ± 0.001	0.002 ± 0.001	0.001 ± 0.0003
(*n *= 5–6/group)						
Endotendon cell shape factor; spindle‐shaped ≤0.5≥ rounded (*n *= 5–6/group)	0.27 ± 0.01	0.22 ± 0.05	0.18 ± 0.02	0.55 ± 0.17	0.61 ± 0.12	0.45 ± 0.05
Tendon collagen type 1, pg/μg total protein* (*n *= 4–8/group)	4.51 ± 0.64	5.90 ± 0.48	5.44 ± 0.79	**7.81 ± 01.30** ^a^	**10.25 ± 0.99** ^a^	**10.02 ± 1.18** ^a^
Tendon collagen type 3, pg/μg total protein* (*n *= 4–8/group)	0.25 ± 0.02	0.23 ± 0.03	0.19 ± 0.02	0.18 ± 0.04	0.26 ± 0.01	0.24 ± 0.04
Myofiber diameters (mcm)	37.53 ± 2.60	37.38 ± 6.27	37.73 ± 2.86	35.26 ± 3.60	37.59 ± 1.89	39.03 ± 4.21
(*n *= 5–6/group)						

FRC, food‐restricted control; HRHF, high repetition high force; IgG, immunoglobulin G; NT, not tested.

Significant changes are bolded.

* Enzyme‐linked immunosorbent assay.

^a^ *p *< 0.05 and ^aa^ *p *< 0.01, compared with matched FRC group.

^b^ *p *< 0.05 and ^bb^ *p *< 0.01, compared with untreated HRHF rats.

^c^ *p *< 0.05 and ^cc^ *p* < 0.01, compared with HRHF + hIgG rats.

### Reductions in HRHF‐Induced Increases in Muscle CCN2

We examined using Western blot if FG‐3019 treatment affected the ability to immunochemically detect CCN2, using tissues from control rats since CCN2 levels should be similar. No differences in CCN2 levels were seen in tendons and serum of FRC versus FRC + FG‐3019 rats (Supplementary Fig. 2).

**Figure 4:**
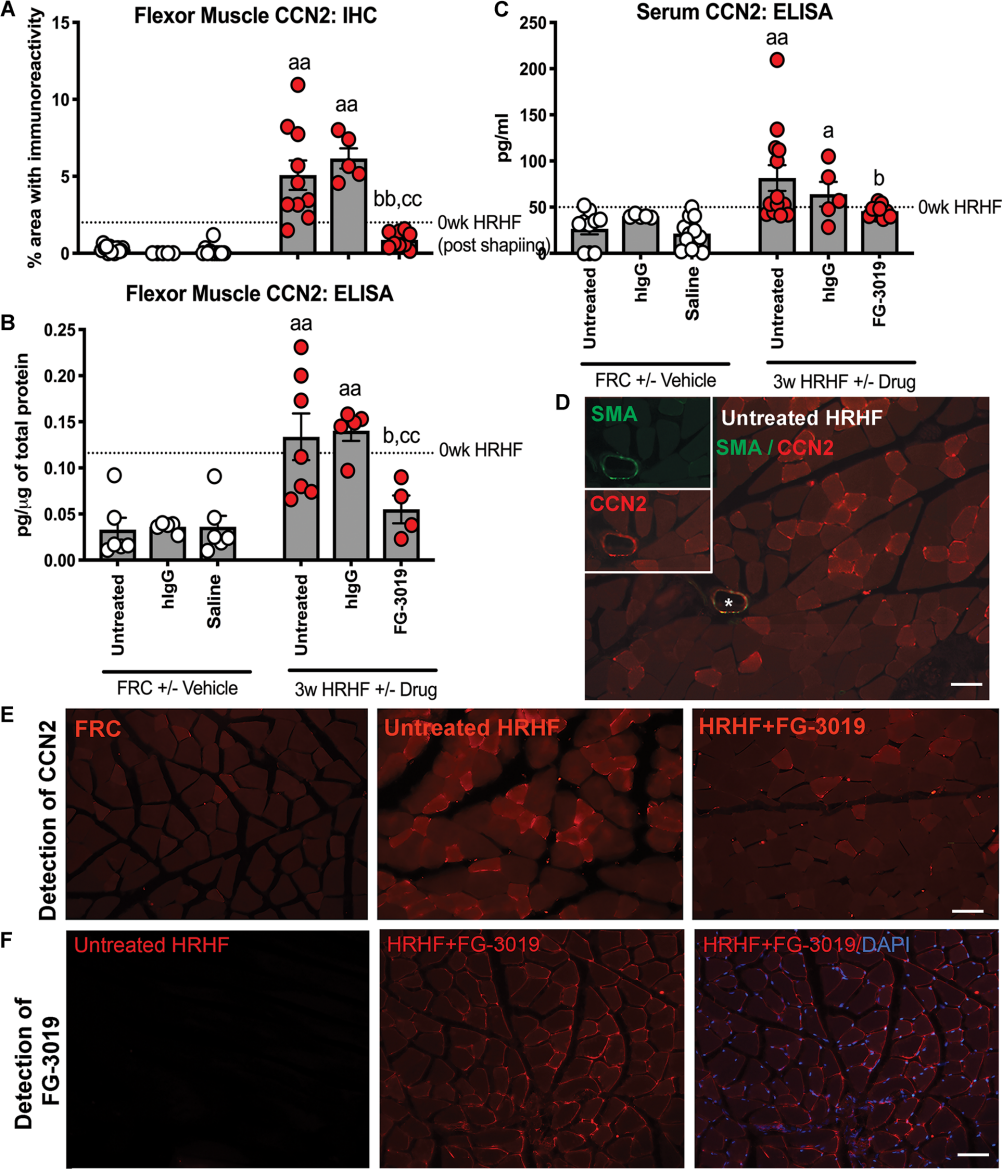
Cellular communication network factor 2 (CCN2) in flexor digitorum muscles. (A–C) Immunoexpression and enzyme‐linked immunosorbent assay (ELISA) detected levels in muscles and serum. (D) An untreated 3‐week high repetition high force (HRHF) muscle probed for both SMA and CCN2 immunoexpression. Only the arteriole shown in the insets shows co‐localization. (E) Location of CCN2 immunoexpression in muscle cross‐sections. (F) Anti‐human IgG detection of the FG‐3019 agent in muscles of HRHF + FG‐3019 rats; staining absent in untreated HRHF rats. a: *p* < 0.05 and aa: *p* < 0.01, compared with matched FRC group; b: *p* < 0.05 and bb: *p* < 0.01, compared with HRHF rats; cc: *p* < 0.01, compared with HRHF + hIgG rats. Scale bars = 50 μm. The 0‐week HRHF rat results from Supplementary Table 1 are indicated with dotted lines [Color figure can be viewed at wileyonlinelibrary.com]

Then, we examined muscles of 0‐week HRHF rats to determine the effects of shaping for 5 weeks to high force pulling, and observed increased CCN2 (immunohistochemically and ELISA assayed) in muscles, compared with FRC rats (*p* < 0.05 each, Supplementary Table 1 and Fig. 4). We next assayed 3‐week HRHF rat muscles, and observed increased CCN2 in HRHF and HRHF + hIgG muscles, compared with FRC and FRC + hIgG rats (Fig. 4A and B). FG‐3019 treatment reduced these increases. CCN2 was detectable in serum, which showed similar responses as muscles (Fig. 4C). Increased CCN2 in untreated HRHF muscles was evident within myofibers and in small cells on their perimeter, relative to FRC and HRHF + FG‐3019 muscles (Fig. 4E), but not in association with αSMA (Fig. 4D). Using an anti‐human IgG antibody, presence of the human FG‐3019 agent was detected in small cells on the perimeter of myofibers in HRHF + FG‐3019 rat muscles, yet not in untreated HRHF rats (Fig. 4F).

**Figure 5:**
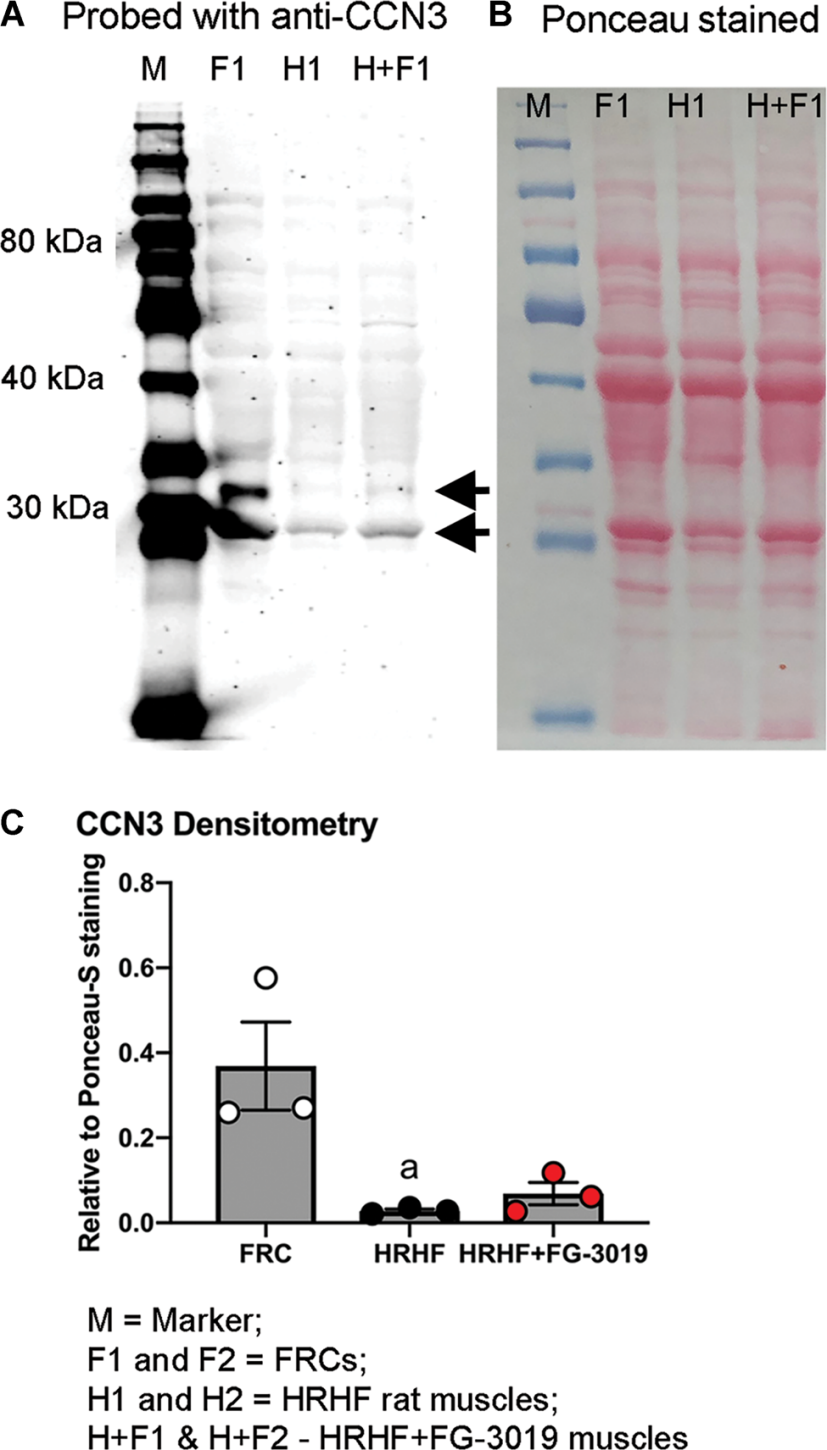
Anti‐CCN3/NOV probed Western blots showing truncated forms of CCN3 with approximate molecular weights of 32 and 28 kDa (arrows). (A) Western blot showing flexor digitorum muscle samples from food‐restricted control rats (F; FRC), untreated high repetition high force rats (H; HRHF), and HRHF rats treated with the FG‐3019 agent (H + F; HRHF + FG‐3019). (B) Ponceau‐S red staining of same membrane shown in panel A. (C) Densitometry results in which CCN3 bands were compared with the total protein loaded per lane, determined from Ponceau‐S red stained membranes. Gels were repeated until three different samples per group were assayed. a: *p* < 0.05, compared with FRC levels [Color figure can be viewed at wileyonlinelibrary.com]

We also examined the effects of task and treatment on CCN1 and CCN3. CCN1 was unchanged with task or treatment (Supplementary Fig. 3). while CCN3 was downregulated in both HRHF and HRHF + FG‐3019 rats, significantly so in HRHF rat muscles, compared with FRCs (Fig. 5).

### Reductions in HRHF‐Induced Increases in Muscle TGFβ1

**Figure 6:**
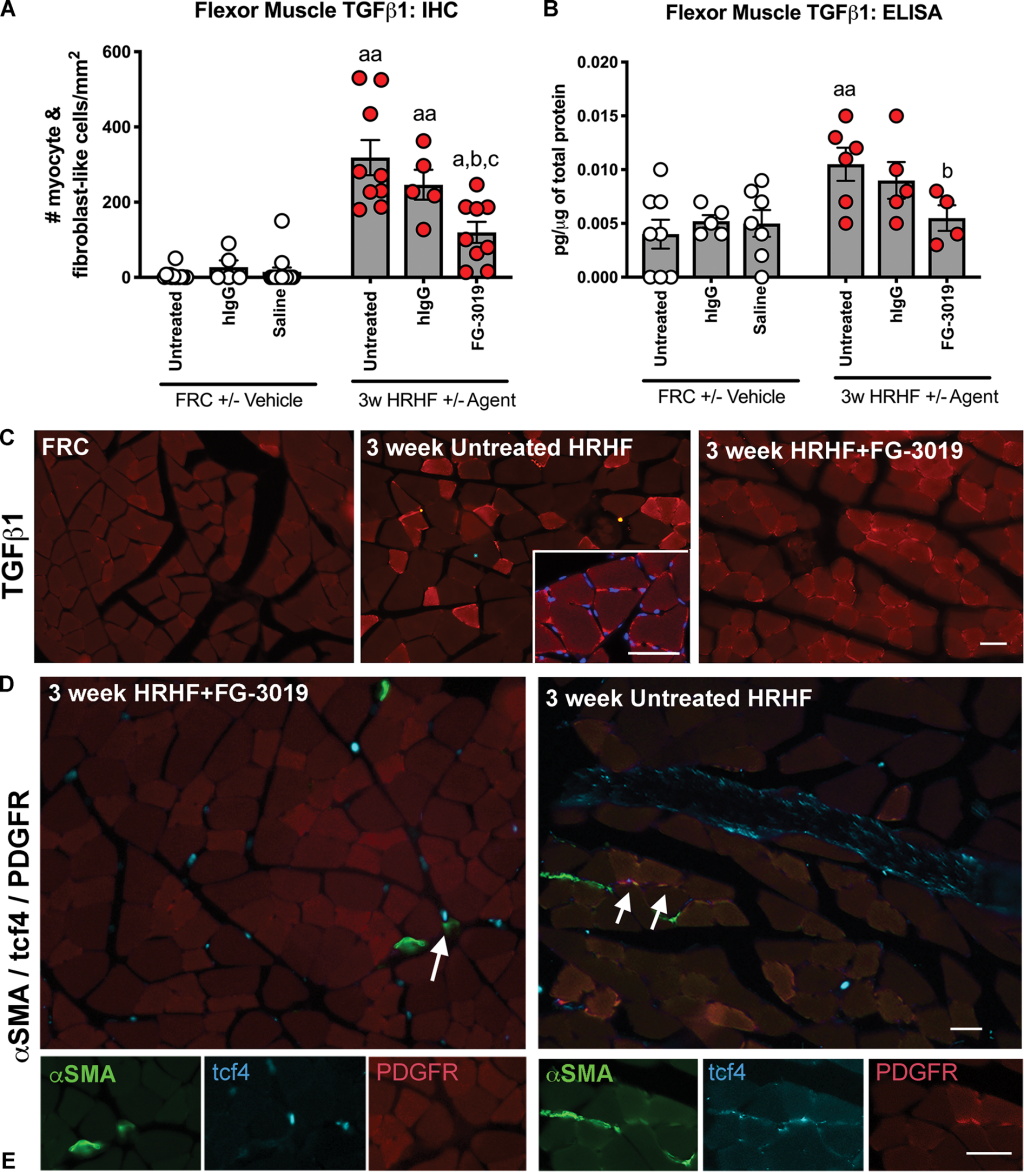
Transforming growth factor β1 (TGFβ1) in flexor digitorum muscles. (A) and (B) Immunoexpression and enzyme‐linked immunosorbent assay (ELISA) detected levels. a: *p* < 0.05 and aa: *p* < 0.01, compared with matched food‐restricted control (FRC) group; b: *p* < 0.05, compared with high repetition high force (HRHF) rats; c: *p* < 0.05, compared with HRHF + hIgG rats. (C) Location of TGFβ1 immunoexpression in muscle cross‐sections. (D) Presence and location of αSMA^+^/tcf4^+^/PDGFR^+^and αSMA^+^/tcf4^+^/PDGFR^−^ cells in HRHF + FG‐3019 and HRHF rat muscles. A few triple‐labeled cells were found on the perimeter of myofibers in 3‐week HRHF rat muscles (arrows). These cells are shown at higher magnification in (E), right side. Left side of (E) shows examples of αSMA^+^/tcf4^+^/PDGFR^−^ cells (i.e., double labeled only) in the endomysium. Scale bars = 50 μm [Color figure can be viewed at wileyonlinelibrary.com]

TGFβ1 immunostaining and levels increased in muscles of HRHF and HRHF + hIgG rats, compared with matched FRCs, and decreased in HRHF + FG‐3019 rats, compared with HRHF rats (Fig. 6A and B). Increased TGFβ1 was seen in small cells on the perimeter of myofibers and in the cytoplasm of some myofibers (Fig. 6C).

Muscle myofibroblast numbers were examined by triple‐labeling with αSMA, tcf4, and PDGFR.^21^ Small but significant increases in αSMA^+^/tcf4^+^/PDGFR^+^ triple‐labeled cells were seen at the edges of myofibers in untreated HRHF rat muscles, compared with HRHF + FG‐3019 and FRC rat muscles (Fig. 6D and E; Table 1). HRHF + FG‐3019 also showed presence of αSMA^+^/tcf4^+^, yet PDGFR cells in the endomysium (Fig. 6C and E), at similar levels as untreated HRHF rat muscles, although more than in FRC rat (data not shown).

### Improved HRHF‐Induced Epitendon Changes

**Figure 7:**
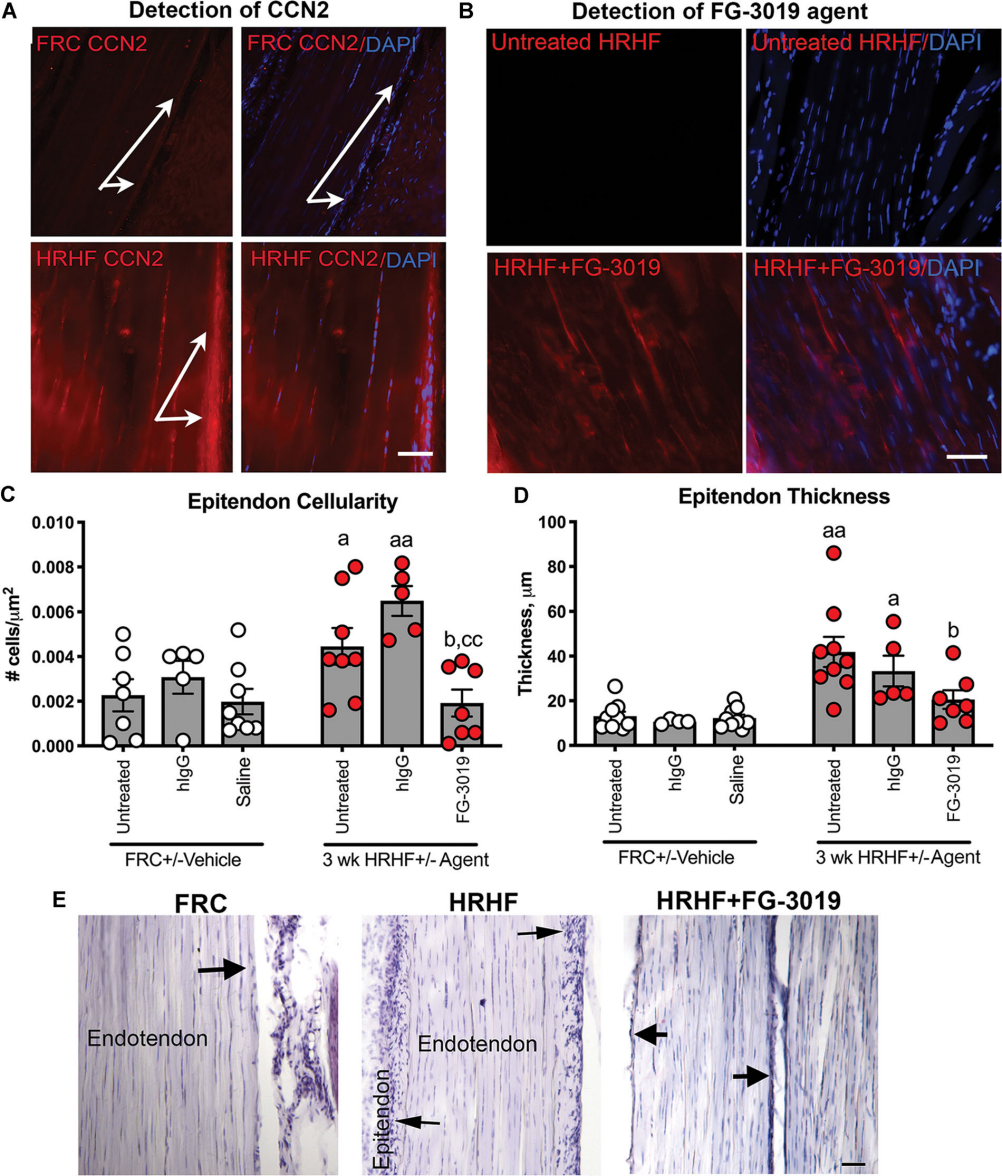
Task‐ and treatment‐induced changes in flexor digitorum tendons at wrist level. (A) Location of cellular communication network factor 2 (CCN2) staining in tendons showing increases in tenocytes and epitendon cells of high repetition high force (HRHF) rats (arrows). (B) Anti‐human immunoglobulin G (IgG) detection of FG‐3019 agent in tendons of HRHF + FG‐3019 rats; staining absent in untreated HRHF rats. (C) and (D) Quantification of epitendon cellularity and thickness. (E) Representative images of hematoxylin and eosin stained tendons. Arrows indicate epitendon region. a: *p* < 0.05 and aa: *p* < 0.01, compared with matched food‐restricted control (FRC) group; b: *p* < 0.05, compared with untreated HRHF rats; cc: *p* < 0.01, compared with HRHF + hIgG rats. Scale bars = 50 μm [Color figure can be viewed at wileyonlinelibrary.com]

CCN2 levels increased in HRHF and HRHF + hIgG tendons, compared with HRHF + FG‐3019, FRC or FRC + hIgG rat tendons (Fig. 7A and Table 1). The FG‐3019 agent was detectable in tendons of HRHF + FG‐3019 rats, yet not in untreated HRHF rats (Fig. 7B). HRHF and HRHF + hIgG rats showed increased epitendon cellularity and thickness, compared with the other groups (Fig. 7C–E), but no endotendon changes (Table 1). Collagen type 1 levels increased in tendons of each task group, compared with FRCs; collagen type 3 levels did not alter with task or treatment (Table 1).

### Reduced HRHF‐Induced Nerve and Dermal Fibrosis

**Figure 8:**
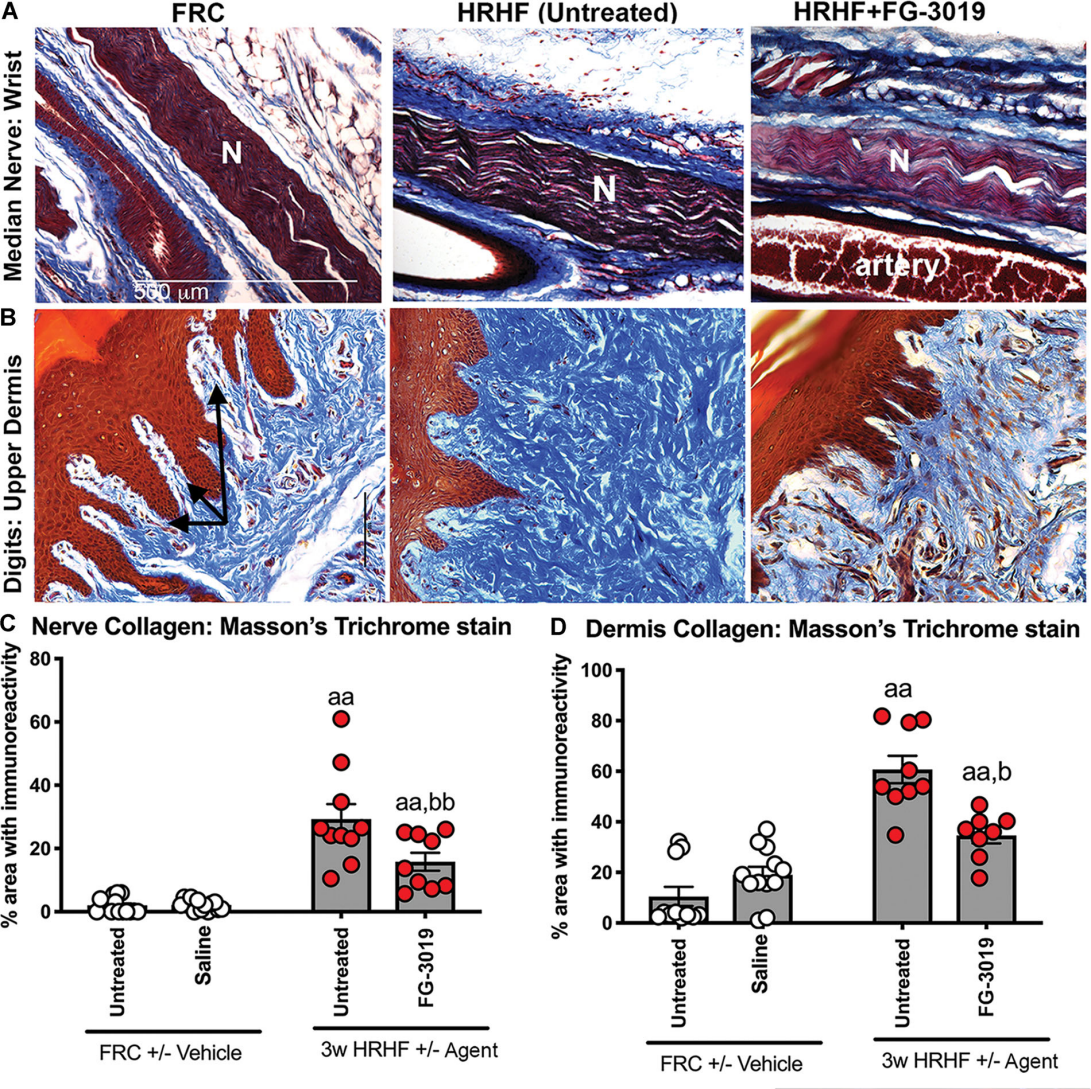
Masson's Trichrome staining around the median nerve branches and in upper dermis of forepaw digits. (A) Masson's trichrome staining with collagen stained blue around the median nerve (N) at wrist level in longitudinal sections. (B) Masson's trichrome staining in the upper dermis. Arrows indicate regions containing nerves within dermal papillae. (C) Quantification of blue‐stained collagen in paraneural regions (i.e., areas surrounding nerve) at wrist level. (D) Quantification of blue‐stained collagen in the upper dermis. aa: *p* < 0.01, compared with matched food‐restricted control (FRC) group; b: *p* < 0.05 and bb: *p *< 0.01, compared with untreated high repetition high force (HRHF) rats [Color figure can be viewed at wileyonlinelibrary.com]

Increased collagen deposition (Masson's Trichrome staining) was observed around median nerves at wrist level and in the dermis of forepaw digits in HRHF rats, compared with FRC and HRHF + FG‐3019 rats (Fig. 8A and B). Increased collagen was also evident in dermal papillae regions containing nerves (Fig. 8B). Quantification confirmed these observations (Fig. 8C and D).

### Correlation of Collagen With Behavioral Outcomes

Pearson's *r* tests (Table 2) showed significant inverse relationships between (i) reaches per minute and % collagen in muscles and collagen levels in tendons, (ii) voluntary grasp force and collagen staining and levels in muscle, and (iii) reflexive grip strength and collagen in all tissues examined. Spearman's *r* tests showed increasing forepaw mechanical sensitivity with increasing nerve/dermal collagen, epitendon cellularity, and thickness.

**Table 2:** Pearson and Spearman's *r* Correlations Between Behavioral Outcomes and Collagen

Tissue Analyses	Reaches/min (Pearson's *r*)	Grasp Force (Pearson's *r*)	Reflexive Grip Strength (Pearson's *r*)	Forepaw Mechanical Sensitivity (3.92 mN Filament; Spearman's *r*)
Muscle: % area collagen type 1 (immunohistochemistry)	***r* = −0.58, *p* = 0.004**	***r* = **−**0.46, *p* = 0.030**	***r* = **−**0.44, *p* = 0.002**	*r *= −0.06, *p* = 0.79
Muscle collagen type 1 (ELISA)	*r* = −0.39, *p* = 0.10	***r* = **−**0.56, *p* = 0.01**	***r* = **−**0.43, *p* = 0.002**	*r* = 0.16, *p* = 0.49
Tendon collagen type 1 (ELISA)	***r *= **−**0.75, *p* = 0.002**	*r* = −0.37, *p* = 0.19	***r* = **−**0.48, *p* = 0.01**	*r* = 0.63, *p* = 0.08
Epitendon: no. of cells/μm^2^	*r* = −0.34, *p* = 0.20	*r* = −0.24, *p* = 0.27	*r *= 0.23, *p* = 0.24	*r* = 0.39, *p* = 0.02
Epitendon: thickness (μm)	*r* = −0.40, *p *= 0.12	*r* = −0.35, *p* = 0.18	*r *= −0.13, *p* = 0.52	***r* = 0.44, *p* = 0.01**
Nerve: % area collagen staining around median nerve at wrist	*r* = −0.39, *p* = 0.09	*r *= −0.20, *p* = 0.39	***r* = **−**0.46, *p* = 0.002**	***r* = 0.50, *p* = 0.01**
Dermis: % area collagen staining in digits’ upper dermis	*r* = −0.33, *p* = 0.19	*r *= −0.38, *p* = 0.139	***r* = **−**0.58, *p* = 0.001**	***r *= 0.68, *p* = 0.001**

ELISA, enzyme‐linked immunosorbent assay.

Significant moderate to strong correlations are bolded.

### Reduced HRHF‐Induced Inflammation

CD68^+^ cells were counted in flexor digitorum muscles and median nerves. Increases were observed in HRHF rats, compared with HRHF + FG‐3019 and FRC rats (Table 3). CCL2 and CXCL10 increased in serum of HRHF rats, compared with FRCs, as did IL‐10, an anti‐inflammatory cytokine (Table 3). Their levels were reduced in HRHF + FG‐3019 rats. Serum levels of CCL3, CXCL2, CXCL5, and IL‐18 were not altered (Table 3).

**Table 3:** Inflammatory Responses in Flexor Digitorum Muscle, Median Nerve, and Serum

Analyte and Tissue	FRC/FRC + Saline (*n* = 5–16)	3‐Week HRHF Untreated (*n* = 6–12)	3‐Week HRHF + FG‐3019 (*n* = 4–9)
Muscle CD68‐immunopositive cells, no. of cells/mm^2^ (*n *= 7–11/group)	0.20 ± 0.08	**0.62 ± 0.06^aa^**	**0.33 ± 0.03^bb^**

Nerve CD68‐immunopositive cells, no. of cells/mm^2^ (*n *= 9–16/group)	6.01 ± 0.88	**20.91 ± 2.92^aa^**	**11.02 ± 2.65** ^b^

Serum CCL2/MCP‐1, pg/ml (*n* = 4–6/group)	92.85 ± 58.06	**494.0 ± 52.91^aa^**	**156.2 ± 68.39^bb^**
Serum CCL3/MIP1a, pg/ml (*n* = 4–6/group)	13.6 ± 2.43	8.07 ± 2.03	9.12 ± 1.01
Serum CXCL2/MIP2, pg/ml (*n* = 4–6/group)	10.62 ± 2.77	9.25 ± 3.89	10.74 ± 3.24
Serum CXCL5/LIX, pg/ml (*n* = 4–6/group)	778.5 ± 93.42	1069.0 ± 127.40	874.4 ± 60.20
Serum CXCL10/IP‐10, pg/ml (*n* = 4–6/group)	172.4 ± 33.24	**345.8 ± 65.80** ^a^	**120.5 ± 12.05** ^b^
Serum IL‐10, pg/ml (*n* = 4–6/group)	0.00 ± 0.00	**112.30 ± 45.41** ^a^	**1.48 ± 1.47** ^b^
Serum IL‐18, pg/ml (*n* = 4–6/group)	20.76 ± 6.16	20.16 ± 5.22	12.82 ± 7.08

FRC, food‐restricted control; HRHF, high repetition high force; IgG, immunoglobulin G.

Significant changes are bolded.

^a^ *p* < 0.05 and ^aa^ *p *< 0.01, compared with matched FRC group.

^b^ *p *< 0.05 and *p *< 0.01, compared with untreated HRHF rats.

### Neither Task Nor Treatment Altered Myofiber Diameters, PAX7, TUNEL, MMP, or ERK

There were no effects of task or treatment in muscles on: mean myofiber diameters (Table 1); numbers of PAX7^+^ or TUNEL^+^ cells in muscles, tendons or nerves (data not shown); muscle MMP2 or MMP9 activity (Supplementary Fig. 4); pERK or total ERK levels (Supplementary Fig. 5).

## DISCUSSION

We previously reported muscle, tendon, and median nerve fibrosis, characterized by increased tissue levels of collagen type 1, CCN2, and TGFβ1 in rats performing the HRHF lever‐pulling task for 6–18 weeks.^8^, ^17^, ^22^, ^23^ The initial 5–6 week shaping period in which rats learn to pull the lever bar at high force levels also enhances CCN2 and TGFβ1 production, relative to control levels^17^, ^19^ (results confirmed and expanded here to show increased collagen type 1 production). Therefore, we chose an antibody that blocks CCN2 signaling (FG‐3019),^14^, ^24^ since CCN2 plays essential roles in mediating excessive matrix deposition in overexpression experiments.^13^, ^25^ We administered this agent in weeks 2 and 3 of HRHF performance to determine if CCN2 contributes to the progression of fibrosis in this model. We found here that FG‐3019 treatment reduced: collagen deposition in multiple tissues (skeletal muscle, dermis, around nerves, and serum), CCN2 production in multiple tissues (muscles, tendons, and serum), TGFβ1 production in muscles, and epitendon cellularity and thickening, each induced by shaping and then performance of the HRHF task for 3 weeks. These reduced tissue changes were associated with improvement in sensorimotor declines.

Rest, ice, compression, and elevation are used to treat acute injuries, but have proved less effective for chronic musculoskeletal disorders. Surgical release or steroid injections are common treatments for fibrotic nerve compression, yet are also not consistently effective.^26^ We postulate that the multi‐tissue involvement of these disorders hinders the effectiveness of interventions focused on improving only one involved tissue. Nonsteroidal anti‐inflammatory drugs (NSAIDS) are also commonly used treatments for acute and chronic musculoskeletal pain,^27^, ^28^ yet are also not completely effective^29^ and may reduce tissue healing.^30^ NSAIDs may fail because they treat algesic symptoms but not the key underlying cause, which we and others believe are fibrotic adhesions tethering tissues and compressing nerves.^9^, ^31^ The FG‐3019 antibody used here targets domain II of CCN2, the vWC domain, located within the amino‐terminal half of the full‐length protein.^4^, ^32^ This domain is thought to stimulate collagen synthesis and bind proteoglycans in the extracellular matrix (other specific associations are being clarified).^4^ Systemic administration of FG‐3019 was used to block fibrogenic processes occurring in all upper extremity tissues involved in performing this chronic repetitive high force task, since collagen production increases in skeletal muscle, tendons, dermis, and extraneural connective tissues, and CCN2 increases in myofibers and small cells on their perimeter, as well as tenocytes, epitendon, mast, and Schwann cells.^8^, ^23^ The effects of blocking other domains of CCN2, such as domains III and IV shown to elicit cell signaling activity,^4^ may also reduce the observed multi‐tissue fibrosis, although this remains to be tested since to our knowledge no antibodies exist that block only specific domain functions.

An early treatment timepoint was chosen to tease out whether CCN2 plays a key role in the progression of fibrosis in this chronic overuse model, or if persistent mechanical loading alone increases collagen production through TGFβ1 signaling pathways independent of CCN2.^33^ As indicated above, increased muscle levels of CCN2 and TGFβ1 are already present in 0‐week HRHF rats,^17^ yet, indices of central sensitization (i.e., spinal cord changes associated with chronic pain) are not detectable until HRHF week 6.^34^ This earlier timepoint allowed us to avoid central nervous system changes that would confound interpretation of behavioral findings.

Sensorimotor behavioral declines in untreated HRHF rats suggest they have either discomfort or reduced tissue function. Fibrosis within and around tissues can reduce dynamic tissue biomechanical function (muscle fibrosis can biomechanically reduce muscle contractions^35^, ^36^). Improved reach rate, voluntary grasp force and reflexive grip strength with FG‐3019 treatment could be due to the reduced tissue collagen, an idea supported by the inverse relationship between motor function and tissue collagen. Fibrotic compression of median nerves in the carpal tunnel leads to reduced nerve conduction, degraded myelin and neuritis.^23^ FG‐3019 treatment of HRHF task rats improved their forepaw mechanical allodynia, in parallel with reduced collagen around nerves at the wrist and forepaw digits. Short‐term FG‐3019 treatment also improves function in irradiated mouse lungs and reduce messenger RNA (mRNA) expression of fibrogenic‐related factors and extracellular matrix production.^37^, ^38^ It has been shown to reduce skeletal muscle fibrosis and improve muscle performance in a mouse model of amyotrophic lateral sclerosis^39^ and a mdx mouse model,^12^ and lung function in human patients with idiopathic pulmonary fibrosis.^14^

Although the use of a targeted monoclonal antibody to a protein has the potential to reduce the ability to detect said protein in tissues, we did not observe differences in CCN2 levels in tendons and serum of untreated controls versus controls treated with FG‐3019. This may be because tissues were collected 5 days after its last injection. Although this should be considered cautiously due to this aforementioned limitation, CCN2 levels were reduced in tissues of FG‐3019‐treated HRHF rats. This finding matches results of an irradiation‐induced lung fibrosis study,^37^ although differing from another showing that FG‐3019 has no effect on skeletal muscle levels of CCN2.^39^ CCN2 levels and signaling responses are known to be context dependent, vary with cell type and matrix in which it is functioning, and environmental stimulants.^40^

We examined muscles for two other members of the CCN family, CCN1/Cyrc61 and CCN3/Nov. A truncated form of CCN1 was observed (~32 kDa), close in molecular weight to the ~28 kDa form representing its NH_2_‐terminal end.^41^, ^42^ CCN1 shows rapid and transient alterations with mechanical loading (<1–6 h).^7^, ^43^, ^44^ However, in this study, muscle CCN1 levels did not alter with task or treatment, likely because tissues were not collected until 36 h after the last task session in order to avoid activity‐induced changes in inflammatory cytokines. This later collection point is also the most probable reason why no changes were observed in MMP2 and pERK levels. CCN1 has a multimodular structure (as do CCN2 and CCN3) that is thought to be the underlying means by which it exhibits diverse activities dependent on environmental context.^42^ Truncated forms of CCN3 were also observed (~32 and 28 kDa), matching the molecular weight of previously identified truncated forms of CCN3.^45^, ^46^ CCN3 is anti‐fibrogenic, antagonizes the fibrogenic effects of CCN2, and has an inverse expression pattern than CCN2.^5^, ^6^ Its downregulation in task animals when CCN2 levels are increasing warrants further examination in fibrogenic musculoskeletal disorders since provision of CCN3 or derived peptides has been postulated as a viable anti‐fibrotic treatment for diabetic nephropathy.^6^

TGFβ1 levels increase in muscles of HRHF rats after shaping and remained increased with continued performance of the HRHF task for up to 18 weeks.^8^, ^17^ It is well known that TGFβ1 is a potent inducer of CCN2 production.^47^ Yet, in the context of this model, blocking CCN2 reduced TGFβ1 production. This agrees with results of a study of diabetic rats where inhibition of CTGF gene expression inhibited TGFβ2 in the retina.^48^ Since mechanical loading and increased TGFβ1 levels are thought to increase myofibroblast numbers in muscles, we examined the flexor digitorum muscles for αSMA^+^/tcf4^+^/PDGFR^+^ triple‐labeled cells.^21^ Only small numbers were seen at the edges of myofibers in untreated HRHF rat muscles (Table 1). These numbers were several fold lower than reported previously in mdx mutant mouse studies.^21^ Thus, although the increases observed in HRHF rat muscles relative to the other groups were statistically significant, this increase is likely not biologically relevant in the context of this study.

FG‐3019 treatment also reduced epitendon fibroblast proliferation and thickening, known to increase in humans and animals with repetitive overuse.^22^, ^49^ The latter findings match those showing decreased fibroblast and mesothelioma cell line proliferation after FG‐3019 treatment.^50^ However, in that study, FG‐3019 treatment also increased cell apoptosis, a response not observed in this study.

Effects of CCN2 on immune cell infiltrates and inflammatory cytokines that contribute to fibrogenic responses are largely unexplored. We were unable to separate out effects of the FG‐3019 agent on inflammation versus those produced by fibrotic tethering of tissues in this in vivo model. What we can say is that CD68^+^ macrophage numbers and serum levels of several chemokines were reduced in HRHF + FG‐3019 relative to HRHF rats. A 2‐week treatment with FG‐3019 reduced macrophage gene signatures and mRNA levels of CCL2 and CXCL10 in mice with radiation therapy‐induced lung injury and fibrosis.^37^ FG‐3019 pretreatment prevented leukocyte influx into lungs after irradiation, while post‐treatment reversed the influx of mast cells and macrophages into lungs when the antibody administration begins 16 weeks after the irradiation.^38^ Inhibition of CCN2 also reduced CD45^+^ leukocytes in an Angiotensin II‐induced mouse model of skin fibrosis.^33^ Although more studies are needed, CCN2 may be a key modulator of inflammatory responses.

We have several limitations. We are unable to tease out inflammatory versus collagen deposition‐induced changes, since these changes occur simultaneously during early phases of our model.^17^ We postulate that inflammation and fibrosis are interrelated in a fibrotic‐inflammatory complex. Perhaps the strength of the FG‐3019 agent was its ability to ameliorate both responses. We cannot address downstream signaling changes in this in vivo model, if we also hope to avoid activity‐induced changes by collecting tissues at 36 h after the last task session. Lastly, we assessed effects of FG‐3019 only in early fibrogenic processes. Studies are nearly complete examining its abilities at reversing established tissue fibrosis in this model.

In conclusion, we evaluated for the first time, the effectiveness of FG‐3019 treatment in reducing the early progression of fibrosis in an operant model of overuse‐induced musculoskeletal disorders. Our findings indicate that CCN2 and its downstream product, collagen, increase early with performance of highly repetitive and forceful tasks. We found that systemic injections of a human monoclonal antibody directed against CCN2 reduced the progression of multi‐tissue fibrosis. Importantly, sensorimotor declines improved, supporting our hypothesis that these behavioral declines are the result of tissue tethering and compression. The effectiveness of this agent in our model of overuse‐induced musculoskeletal disorders shows promise for the treatment of related disorders in human subjects.

## ACKNOWLEDGMENTS

Research reported in this publication was supported by the National Institute of Arthritis and Musculoskeletal and Skin Diseases of the National Institutes of Health under Award Number AR056019, and by American Society of Bone and Mineral Research Gap Award 1025. The content is solely the responsibility of the authors and does not necessarily represent the official views of the National Institutes of Health.

## Supporting information

Supporting information

Supporting information

Supporting information

Supporting information

Supporting information

Supporting information

Supporting information
