# Supplementary material for: Blocking CCN2 Reduces Progression of Sensorimotor Declines and Fibrosis in a Rat Model of Chronic Repetitive Overuse
Source: J Orthop Res. 2019 Jun 20;37(9):2004–18. doi: 10.1002/jor.24337 (PMC6688947; doi:10.1002/jor.24337)
Supplement: Supplementary file 1 — Supporting information [file JOR-37-2004-s001.docx]

Supplemental Table 1. Effect of shaping to high force lever pulling across a 5 weeks.

Significant findings are bolded.

| **Analyte and Tissue** | **FRC untreated**  (n=10) | **0-week HRHF (post shaping)**  (n=5-10) |
| --- | --- | --- |
| Muscle Collagen type 1, % immunoexpression | 2.47±0.59 | **9.50±2.81^a^** |
| Muscle Collagen type 1, pg/μg total protein^2^ | 0.74±0.06 | **1.49±0.36^a^** |
| Serum Collagen type 1, pg/ml serum^2^ | 6.78±1.41 | 10.77±2.08 |
| Muscle CCN2, % immunoexpression | 0.23±0.05 | **2.10±0.06^a^** |
| Muscle CCN2, pg/μg total protein^2^ | 0.04±0.01 | **1.12±0.06^a^** |
| Serum CCN2, pg/ml serum^2^ | 26.65±6.17 | 49.49±32.64 |

^1^ % area with immunoexpression; ^2^ELISA

^a^p<0.05, compared to FRC rats
