# Supplementary material for: Blocking CCN2 Reduces Progression of Sensorimotor Declines and Fibrosis in a Rat Model of Chronic Repetitive Overuse
Source: J Orthop Res. 2019 Jun 20;37(9):2004–18. doi: 10.1002/jor.24337 (PMC6688947; doi:10.1002/jor.24337)
Supplement: Supplementary file 2 — Supporting information [file JOR-37-2004-s002.docx]

Supplemental Materials: Detailed Methods

DETAILED METHODS

*Overview of Animals*

Experiments were approved by the Temple University Institutional Animal Care and Use Committee in compliance with NIH guidelines for the humane care and use of laboratory animals. Studies were conducted on young adult (2.5 months of age at onset), female, Sprague-Dawley rats (Charles Rivers, Wilmington, MA). Rats were housed individually in standard rat cages (ventilated and with hardwood chip rodent bedding) in a central animal facility in a 12- hour light: 12-hour dark cycle with free access to water. Rats used in the study were first acclimated to the animal facility for 1 week before a second week of handling only, and the onset of food restriction to 5% less than the weights of age-matched, normal control rats with free-access-to-food; these latter rats were used for weight comparison purposes only.

Task rats were randomly chosen. These rats first underwent an initial shaping period in operant behavioral chambers for 5 weeks to learn the high force lever pulling task, before going on to perform the HRHF task for 3 weeks. HRHF rats were randomly divided into subcohorts that were either left untreated or treated with an anti-CCN2 agent (FG-3019) or a human IgG (hIgG). Reach limbs were examined from untreated HRHF, HRHF+hIgG, and HRHF+FG-3019 rats (n=17, 10 and 13, respectively). Remaining food-restricted rats were maintained as food-restricted controls (FRC) that were untreated or treated with FG-3019, hIgG or saline (again, randomly chosen into these subcohorts). Forelimb tissues were collected from 29 FRC, 22 FRC+Saline, 5 FRC+hIgG, and 5 FRC+FG-3019 rats, bilaterally. A larger number of FRC rats than experimental rats were used to accommodate testing of improved gelatin zymography, tissue processing refinement, and antibody specificity. FRC rats received similar amounts of rat chow and food reward pellets as HRHF rats. Muscle and serum tissues were also examined from seventeen 0-week HRHF rats (euthanized after the shaping period).

All rats were weighed twice per week, provided regular rat chow daily (PicoLab Rodent Diet #5053, Lab Diet, Durham, NC), in addition to food reward pellets (a mix of Banana sucrose and Chocolate dustless precision pellets; # F0024 and #F0299, 45 mg, Bio-Serv, Flemington, NJ), and allowed to gain weight over the course of the experiment, since they were young adult rats at onset of experiments (Supplemental Figure 1). FRC rats received similar amounts of rat chow and food reward pellets as HRHF rats. All rats were handled at least twice per week and provided cage enrichment toys that included chew bones, tunnels and paper twists (Diamond Twists, Teklad #7979C.CS, Envigo, South Easton, MA).

To reduce illness-related confounders, additional sentinel rats were examined for presence of illnesses as part of regular veterinary care (none were detected).

*Pharmacological Treatments*

Subcohorts of HRHF task rats were either left untreated, or were treated in task weeks 2 and 3 with a human anti-CCN2 monoclonal antibody (FG-3019, a gift from FibroGen, Inc., San Francisco, CA; 40 mg/kg body wt, i.p.; 2x per wk for 2 wks); or human IgG (hIgG, a gift from FibroGen, Inc.; 50 μl/injection, i.p.; 2x per wk for 2 wks). FRC rats were either untreated or treated similarly with the same drugs or the vehicle, saline (100 μl saline, i.p., 2x/wk). Mode of injection (i.p.) and doses were chosen based on company’s recommendations.

*Behavioral Apparatuses, Shaping, and Task Regimen*

Sixteen custom-designed operant behavioral chambers were used in which rats performed an operant reaching and lever pulling task, as previously described ^1^. Briefly, animals reached through a shoulder height portal to pull on a force lever bar attached to a force transducer (Futek Advanced Sensor Technology, Irvine, CA) located outside the chamber wall and attached to a load cell. This bar was attached to a tension-compression load cell (#LSB200, Futek) interfaced with a strain-gauge amplifier (#CSG110, Futek). The load cell signal was sampled digitally at 100 Hz using custom-written Force Lever software (#ENV-118M, Med Associates, St. Albans, VT). Task rats were shaped in these operant chambers across a 5 week shaping period to learn a reaching and lever-pulling task at high force loads at no specified reach rates (ramping upwards from naïve for 10 min/day, 5 days/wk). Rats went on to perform a HRHF reaching and lever-pulling task for 3 weeks (48% of their maximum pulling force (1.39 Newtons), 4 reaches/min, 2 hrs/day, in 30 min intervals with 1.5 hr rest breaks between, 3 days/wk), using custom written software that required a specific force pull for a food reward (Med Associates, St. Albans, VT). This occurred on Monday, Wednesdays and Fridays, beginning at 9:30 am. Additional details are as previously published ^2^. Limbs used to reach were recorded each session.

*Behavioral Assays*

HRHF task reach outcomes were recorded continuously during each task session and later extracted into Excel ^3^. For this study, reach rate (number of reaches per minute) and grasp force on the lever bar was calculated in task week 3 for the primary reach limbs of 12 HRHF, 5 HFHF+IgG and 10 HRHF+FG-3019 rats. These data could not be generated for FRC rats, as they did not perform the task.

All HRHF and FRC rats were assayed for grip strength using a grip strength meter (1027SR-D58, Columbus Instruments, Columbus, Ohio). Grip strength was assayed after onset of food restriction, 5 weeks later after training (HRHF week 0), and at the end of HRHF week 3. The test was repeated 5 times/limb. Maximum grip strength per trial is reported. A two-choice temperature place preference test and a temperature testing apparatus (T2CT, Bioseb, France) was used to determine cold aversion ^4^. One plate was a reference plate at 22^o^C while the test plate was adjusted from 22-12°C. Rats were free to choose their preferred position on the plates. Forelimb sensitivity to mechanical probing was assayed at end of HRHF task week 3 using Seemes-Weinstein monofilaments (Stoelting, IL) ^3^. Mean number of limb withdrawal responses out of 10 repeats is reported for each monofilament used. Number of limbs tested per group: HRHF, n=12; HRHF+FG-3019, n=10-12; HRHF+hIgG, n=10; FRC, n=10-25; FRC+Saline, n=10; FRC+hIgG, n=10, and FRC+FG-3019, n=10, with bilateral results included for the latter two groups.

*ELISAs*

Animals were deeply anesthetized with 5% isoflurane in oxygen, and euthanized by cardiac puncture for blood collection using an 18-gauge needle at 36 hr after their last task session. Blood was collected from HRHF rat groups (13 HRHF, 9 HRHF+FG-3019 and 5 HRHF+hIgG), FRC groups (11 FRC, 5 FRC+hIgG, 8 FRC+Saline), and seven 0-week HRHF rats. Collected blood was placed into uncoated 15 ml tubes and kept on ice for 30 min before being centrifuged at 12000 rpm for 20 min at 4^o^C. Serum was harvested and frozen at -80^o^C until assayed using commercially available single-plex ELISA kits for levels of: Collagen type 1 (#LS-F5638, LifeSpan BioSciences, Inc., Seattle, WA), Collagen type 3 (#LS-F23886, LifeSpan BioSciences), connective tissue growth factor (CCN2 CTGF; #024398, USBiological, San Diego, CA), and transforming growth factor beta 1 (TGFβ1; #ADI-900-155, Enzo Life Sciences, Inc, Farmingdale, NY). Serum was also assayed with a multiplex kit for 7 inflammatory cytokines and chemokines (CCL2/MCP-1, CCL3/MIP1a, CXCL2/MIP2, CXCL5/LIX, CXCL10/IP-10, IL-10, IL-18; #LXSARM, R&D Systems, Minneapolis, MN). ELISAs were conducted using manufacturers’ protocols and results analyzed using a microplate reader (Bio-Rad Laboratories, Inc., Hercules, CA) or a multiplex Millipore reader. Each sample was run in duplicate and is reported as pg of protein per ml of serum.

Subsets of forelimb soft tissues of rats were similarly assayed using ELISA. For this, limbs used to reach were collected from ten 0-week HRHF rats, subcohorts of HRHF rats (10 HRHF, 4 HRHF+FG-3019 and 5 HRHF+hIgG), one limb per FRC rat (16 FRC, 5 FRC+hIgG, 10 FRC+Saline, and 5 FRC+FG-3019), the latter removed prior to perfusion of rats with fixative for histological assays described later. For ELISA, flexor digitorum muscles and tendons were separated from bones and each other using a scalpel (the remaining forepaw was removed used for histological assays as described further below). Muscle and tendon samples were rinsed in sterile saline and flash frozen before storage at -80^o^C until use, at which time they were thawed on ice and homogenized in sterile, ice-cold, phosphate-buffered saline (PBS) containing fresh proteinase inhibitors (1 tablet per 25 ml of PBS; cOmplete EDTA free Protease Inhibitor tablets, #5056489001, Sigma-Aldrich, Inc., St. Louis, MO). For this, muscle and tendon samples were homogenized, separately, in 500 ml of the buffer (using a Tekmar Tissuemizer Homogenizer, model SDT-1810). Buffer was increased further to 1000 ml total for muscles. Homogenates were centrifuged at 12000 rpm for 15 min at 4^o^C. Supernatants were aliquoted and stored at -80^o^C until assayed for Collagen type 1, Collagen type 3, CCN2 and TGFβ1, using the same kits as described for serum. ELISAs were conducted as for serum. Muscle and tendon results (pg of protein) were normalized to μg of total protein, determined using a bicinchoninic acid (#3225, BCA Protein assay, Pierce^TM^, Thermo Fisher Scientific, Waltham, MA).

*Gelatin Zymography for MMP2 and MMP9*

Aliquots of muscles lysates were also assayed for matrix metalloproteinase (MMP) 2 and MMP9 activity using gelatin zymography. First, 8% SDS-PAGE gels were prepared with 0.05% gelatin. Supernatants of muscle homogenates were mixed with 6X Laemmli SDS buffer (5 parts sample to 1 part Laemmli buffer) and incubated at room temperature for 10 min, without a reducing agent or heating (i.e., non-reducing conditions). BCA assays were performed as above and 20 μg of total protein per sample was loaded into each well. Purified recombinant rat MMP9 and MMP2 were used as positive controls (#5427-MM-010 and #924-MP-010, respectively; R&D Systems). Novex Tris-Glycine SDS running buffer was used (#LC2675; Invitrogen, Life Technologies, Carlsbad, CA), and the gels run at 125 V. Gels were then washed with dH_2_O, renatured with 2.5% Tween-20 in dH_2_O for 45 min (3 x 15 min each), rinsed with a developing buffer for 30 min (33 ml Tris saline buffer with 5mM CaCl_2_ and 2 μm ZnCl_2_; 6 x 5 min each), and then incubated in this latter buffer for 48 hours. Gels were rinsed in dH_2_O for 24 hours before staining with 0.05% commassie blue R250 (in 200ml methanol, 50 ml acetic acid, 250 ml dH_2_O) for 15 min, and then destained for two hours in 437.5 ml dH_2_O, 37.5 ml acetic acid and 25 ml methanol. After rinsing again in dH_2_O, gels were imaged. The location of gelatinolytic activity was detectable as a clear band against a background of uniform blue staining. All white MMP2 band densities were normalized to the mean of all FRC and FRC+saline results, and these ratios were compared statistically. Gels were repeated until 3 different samples per FRC group were assayed, and 6 different samples per HRHF group were assayed (6 gels total).

*Western blot assays for CCN1, CCN2, CCN3, pERK and total ERK*

# Western blot analyses for CCN1, CCN2, CCN3, phosphorylated (p) ERK 1/2 and total ERK protein expression were performed on muscle lysates. Protein content was measured in duplicate as above (BCA Protein Assay Kit). Laemmli buffer (4X) and 5% beta-mercaptoethanol (BME) were added and samples were heated at 100°C for 5 min, shortly vortexed and spun in a microcentrifuge tube. Equal amounts of protein (20 µg/20 µL) were separated by SDS-PAGE (Mini Gel Tank, Invitrogen).

For detection of CCN1/Cyr61, CCN2 and CCN3, 10% Tris-Glycine SDS gels were pored and used. The gels were run at 125V for approximately 65 minutes. Gels were blotted onto nitrocellulose membranes (1.25 hours at 20V) using Invitrogen equipment. Gels were blocked for 1 hr in 5% nonfat milk in Tris buffered saline (TBS). Membranes were then incubated with primary antibodies, each diluted 1:1000 overnight at 4°C with shaking: 1) CCN1/Cyr61 (anti-CCN1 Pico band antibody, # PB9549, Boster Biological Technology, Pleasanton, CA; 2) CCN2 (anti-CCN2, #sc-14939 (L-20), Santa Cruz Biotechnology, Santa Cruz, CA); and 3) CCN3 (anti-CCN3/NOV, [EPR8781(3)], # ab191425, Abcam, Cambridge, MA). Membranes were washed in TBS with 0.05% Tween and then incubated for 1 hour with appropriate 680 and/or 800-conjugated secondary antibodies (IRDye® Infrared Dyes, LI-COR, Lincoln, NE, USA) diluted 1:5000. Images were obtained using a LI-COR System and analyzed for densitometry using Image J software. For CCN1, CCN2 and CCN3 bands were compared to the total protein loaded per lane, determined for each lane from Ponceau-S stained membranes (Sigma-Aldrich, St. Louis, MO; stained prior to antibody probing). These ratios were compared statistically and graphed. Gels were repeated until 3 different samples per group were assayed (3 gels total).

# For ERK and pERK detection, pre-cast 4-12% Tris-Glycine gels (Novex^TM^ WedgeWell^TM^ 4-12% Tris-Glycine Gels, Invitrogen) were used. The gels were run at 210 V for 35 min. Gels were blotted (Pierce Power Blotter, ThermoFisher Scientific, 25V, 2.5A, 10 min) onto nitrocellulose membranes (#10600002, Amersham^TM^ Protran^TM^ 0.45 µm NC, GE Healthcare Life Science, Germany), blocked for 1 hr in 5% BSA dissolved in TBS with 0.05% Tween-20, and incubated with primary antibodies, each diluted 1:1000 overnight at 4°C with shaking: 1) total Erk1/2 (anti-p44/42 MAPK (total Erk1/2), #137F5, Cell Signaling Technologies, Danvers, MA); and 2) pErk1/2 (anti-Phospho-p44/42 MAPK (pErk1/2; Thr202/Tyr204), #D.133.14.4E, Cell Signaling Technologies). Membranes were washed in TBS with 0.05% Tween and then incubated for 1 hour with appropriate 680 and/or 800-conjugated secondary antibodies (IRDye® Infrared Dyes, LI-COR, Lincoln, NE, USA) diluted 1:5000. Images were obtained using a LI-COR System and analyzed for densitometry using Image J software. Band densities were normalized to an internal control sample loaded onto each gel. Normalized bands of pERK and total ERK were then compared as a ratio (pERK/total ERK). Normalized bands of pERK were also compared to the total protein loaded per lane, determined for each lane from Ponceau-S stained membranes (Sigma-Aldrich, St. Louis, MO; stained prior to antibody probing); these ratios were compared statistically and graphed. Gels were repeated until 4 different samples per group were assayed (4 gels total).

*Ihistochemistry and Immunohistochemistry*

Rats were deeply anesthesized as described above, serum collected and limbs to be used for ELISAs/homogenates removed, before undergoing transcardial perfusion with first saline and then with 4% paraformaldehyde in phosphate buffer (pH 7.4). Reach limbs were collected from subcohorts of HRHF rats (13 HRHF, 9 HRHF+FG-3019 and 5 HRHF+hIgG), at least one limb per FRC rat (16 FRC, 5 FRC+hIgG and 15 FRC+Saline), and ten 0-week HRHF rats. Limbs were postfixed in the same fixative for 48 hours. Soft tissues of the forearm were collected and cryoprotected in 10% sucrose (48 hours) and then 30% sucrose in phosphate buffer (48 hours). A 2.5 mm thick piece of each flexor digitorum muscle was removed using a scalpel from the mid- to proximal end of the muscle mass and placed in cryomolds for crossectional slicing, while the remaining muscle-tendon-nerve mass spanning a region from mid-forearm to the wrist was placed lengthwise in the same cryomold for longitudinal sectioning. These tissues were then frozen at -80^o^C until cryosectioned into 15 μm thick sections and placed onto charged and coated slides (Tissue Path Superfrost Plus Gold Slides, Fisher Scientific, Fair Lawn, NJ) and dried overnight at room temperature, before storage in foil-wrapped slide boxes at -80^o^C until use.

Subsets of muscle-tendon-nerve mass cryosections were stained in batched sets with hematoxylin before coverslipping with 80% glycerol in PBS to prevent shrinking, or TUNEL (#S7165, ApopTag-Red, Millipore, Temecula, CA, using manufacturer’s directions) before coverslipping with 80% glycerol in PBS. Subsets were also immunostained in batched sets for collagen type 1, collagen type 3, CCN2, PAX7, TGFβ1, using previously described methods ^4-6^, before coverslipping with 80% glycerol in PBS. The primary antibodies included were anti-collagen type 1 (#C2456, Sigma, 1:500 dilution in PBS), anti-collagen type 3 (#AB6310; Abcam Laboratories, Hercules, CA, 1:500 dilution in PBS), anti-CCN2 (sc-14939 (L-20), Santa Cruz Biotechnology, Santa Cruz, CA, 1:400 dilution in PBS), anti-PAX7 (#AB199010, Abcam, 1:20 dilution in PBS), and anti-TGFbeta1 (#MAB240, R&D, dilution 1:500 in PBS). Secondary antibodies used included: a goat anti-mouse IgG with an AF488 (green) tag (#AB150117, Abcam), a goat anti-rabbit IgG with a Cy3 (red) tag (#115-165-166, Jackson ImmunoResearch, West Grove, PA), and a goat anti-mouse IgG with a green tag (Alexa Fluor 488, #ab150117, Abcam). Secondary antibodies were all diluted 1:100 in PBS and incubated on sections for 2 hours before washing in PBS. DAPI was used as a nuclear stain (#62246, Thermoscientific; diluted 1: 2000 with PBS for 15 minutes) before coverslipping with 80% glycerol in PBS.

Other subsets of slides that included muscles were triple-labeled. First, with antibodies against alpha smooth muscle actin (αSMA; (# A2547, Sigma, 1:500 dilution in PBS) and platelet derived growth factor receptor (PDGFR, #ab203491, Abcam, 1:250 dilution in PBS) for overnight at room temperature (after first a pepsin digestion using a 1:6 dilution of 12000 units /ml of pepsin in 0.01N HCL for 15 min, and then a 30 min incubation in 10% goat serum). Sections were then washed in PBS (3 times 5 min each). Slides were then incubated with appropriate secondary antibodies for these two primaries: a goat anti-mouse IgG with an AF488 green tag (# AB150117, Abcam) and a goat anti-rabbit with a Cy3 red tag (# 111-165-144, Jackson ImmunoResearch), respectively, each diluted 1:100 in PBS. Next washed slides were incubated with the third antibody against transcription factor 4 (tcf4/TCF7L2 (# ab76151, Abcam, diluted 1:250) for overnight at room temperature, washed, before incubation with a goat anti-rabbit with an AF647 far-red tag that was later pseudocolored after imaging using Photoshop to a pale blue (#111-605-144, Jackson ImmunoResearch). DAPI was used as a nuclear stain before coverslipping with 80% glycerol in PBS.

Additionally, an anti-human IgG tagged with Dylight 650 (#ab96906, Abcam) was used to detect the FG-3019 monoclonal antibody. Slides were first blocked with 10% goat serum plus 0.1% Triton in PBS for 30 minutes at room temperature. The anti-human IgG was diluted 1:200 in PBS, before incubating the slides with antibody overnight at room temperature. After washing three times in PBS, sections on slides were DAPI stained, and then coverslipped with 80% glycerol in PBS. Antibody specificity was determined for the anti-human IgG by staining sections from animals that had not been injected with the FG-3019 agent.

Antibody specificity for collagen type 1, CCN2 and TGFbeta1 was determined by: 1) leaving out the primary antibody, 2) western blot assays to ascertain if the correct molecular weights were detected, and 3) peptide or protein blocking assays; these results have been previously published ^4, 5, 7^. Specificity of TUNEL staining was performed as previously described ^8^.

Forepaw tissues of all reach limbs, including the digits, were postfixed by immersion as described above, and then processed for paraffin embedding, as previously described ^9^. Forepaw tissues were embedded in paraffin, sectioned longitudinally into 5 μm thick sections, and placed onto charged and coated slides. These sections on slides were stored at room temperature until use, at which point they were deparaffinized in xylene and decreasing concentration of ethanols before staining with hematoxylin (Modified Harris Hematoxylin, Thermo Fisher Scientific, Kalamazoo, MI), or Masson’s Trichrome stain, followed by coverslipping with DPX mountant (#06522, Sigma-Aldrich).

*Histomorphometry*

Quantification of the fluorescence immunostaining in the cryosections was performed in batched sets by individuals blinded to group assignment, using upright microscopes with both bright field and epifluorescent features (E800 and E1000, Nikon, Melville, NY) interfaced with digital cameras (Retiga 4000R QImaging Firewire Cameras, Surry, BC Canada), PC computers, and image analysis systems (Bioquant OsteoII or Life Science, Bioquant Image Analysis Corporation, Nashville, TN). In flexor digitorum muscle cryosections, collagen types 1 and 3, and CCN2 immunostaining was quantified in the mid- to proximal ends of the muscle regions that had been cut crossectionally, using a thresholded pixel count, using a 20x objective and previously described methods ^10^. Briefly, a thresholded pixel count is the number of pixels showing immunostaining at or above a selected threshold, divided by the total number of pixels in an area of interest. CCN2 immunostaining was quantified similarly in longitudinal cryosections of flexor digitorum tendons. Numbers of TGFβ1, CD68, PAX7 and TUNEL immunopositive cells were counted in mid- to proximal regions of the flexor digitorum muscle (crossections), in three fields per muscle and rat, using a 20x objective. Numbers of CD68 cells within the median nerve were counted at the level of the wrist in the longitudinally cut cryosections, in three fields per nerve and rat, using a 20x objective. Cell count data is presented as numbers per mm^2^.

Shortest diameters of individual myofibers were assayed in the collagen/DAPI immunostained cross-sectional cryosections of flexor digitorum muscles in 3 fields/muscle, using a 20x microscope objective, with data reported as mean circular mil (1000 circular mil, abbreviated as mcm, equals 0.5067 mm^2^), using Bioquant Life Science histomorphometry software. Epitendon thickness and cellularity (number of cells per mm^2^), as well as endotendon cellularity, were assayed in the flexor digitorum tendons of longitudinally cut cryosections after hematoxylin staining. These tendon counts were performed within the boundaries of the epitendon at the level of the wrist using an irregular region of interest tool of the Bioquant image analysis program. The cell shape factor analysis feature of the Bioquant program was used to assay if endotendon cells were more spindle-shaped versus rounded in these same hematoxylin stained sections (spindle shaped < 0.5, while > 0.05 to 1 indicates a more rounded shape).

Collagen deposition around the median nerve was quantified after Masson’s Trichrome staining (which stains collagen blue) of the paraffin-embedded and longitudinally cut forepaws. The percent area with collagen staining was quantified using a threshold pixel count in the extraneural fascia immediately surrounding nerve branches located at the level of the wrist, in a region of interest that was 20 μm external to the epineurium. Collagen deposition in the upper dermis of the skin of the digits was similarly counted in these same sections.

References for Supplemental Methods:

1. Barbe MF, Gallagher S, Massicotte VS, Tytell M, Popoff SN, Barr-Gillespie AE. The interaction of force and repetition on musculoskeletal and neural tissue responses and sensorimotor behavior in a rat model of work-related musculoskeletal disorders. BMC Musculoskelet Disord 2013;14:303.

2. Barbe MF, Gallagher S, Massicotte VS, Tytell M, Popoff SN, Barr-Gillespie AE. The interaction of force and repetition on musculoskeletal and neural tissue responses and sensorimotor behavior in a rat model of work-related musculoskeletal disorders. BMC Musculoskelet Disord 2013;14:303.

3. Xin DL, Hadrevi J, Elliott ME, et al. Effectiveness of conservative interventions for sickness and pain behaviors induced by a high repetition high force upper extremity task. BMC Neurosci 2017;18:36.

4. Fisher PW, Zhao Y, Rico MC, et al. Increased CCN2, substance P and tissue fibrosis are associated with sensorimotor declines in a rat model of repetitive overuse injury. J Cell Commun Signal 2015:1-18.

5. Abdelmagid SM, Barr AE, Rico M, et al. Performance of repetitive tasks induces decreased grip strength and increased fibrogenic proteins in skeletal muscle: role of force and inflammation. PLoS One 2012;7:e38359.

6. Rani S, Barbe MF, Barr AE, Litvin J. Induction of periostin-like factor and periostin in forearm muscle, tendon, and nerve in an animal model of work-related musculoskeletal disorder. J Histochem Cytochem 2009;57:1061-1073.

7. Gao HG, Fisher PW, Lambi AG, et al. Increased Serum and Musculotendinous Fibrogenic Proteins following Persistent Low-Grade Inflammation in a Rat Model of Long-Term Upper Extremity Overuse. PLoS One 2013;8:e71875.

8. Barbe MF, Massicotte VS, Assari S, et al. Prolonged high force high repetition pulling induces osteocyte apoptosis and trabecular bone loss in distal radius, while low force high repetition pulling induces bone anabolism. Bone 2018;110:267-283.

9. Barr AE, Safadi FF, Gorzelany I, Amin M, Popoff SN, Barbe MF. Repetitive, negligible force reaching in rats induces pathological overloading of upper extremity bones. J Bone Miner Res 2003;18:2023-2032.

10. Al-Shatti T, Barr AE, Safadi FF, Amin M, Barbe MF. Increase in inflammatory cytokines in median nerves in a rat model of repetitive motion injury. J Neuroimmunol 2005;167:13-22.
