# Supplementary figures and images for: Blocking CCN2 Reduces Progression of Sensorimotor Declines and Fibrosis in a Rat Model of Chronic Repetitive Overuse
Source: J Orthop Res. 2019 Jun 20;37(9):2004–18. doi: 10.1002/jor.24337 (PMC6688947; doi:10.1002/jor.24337)

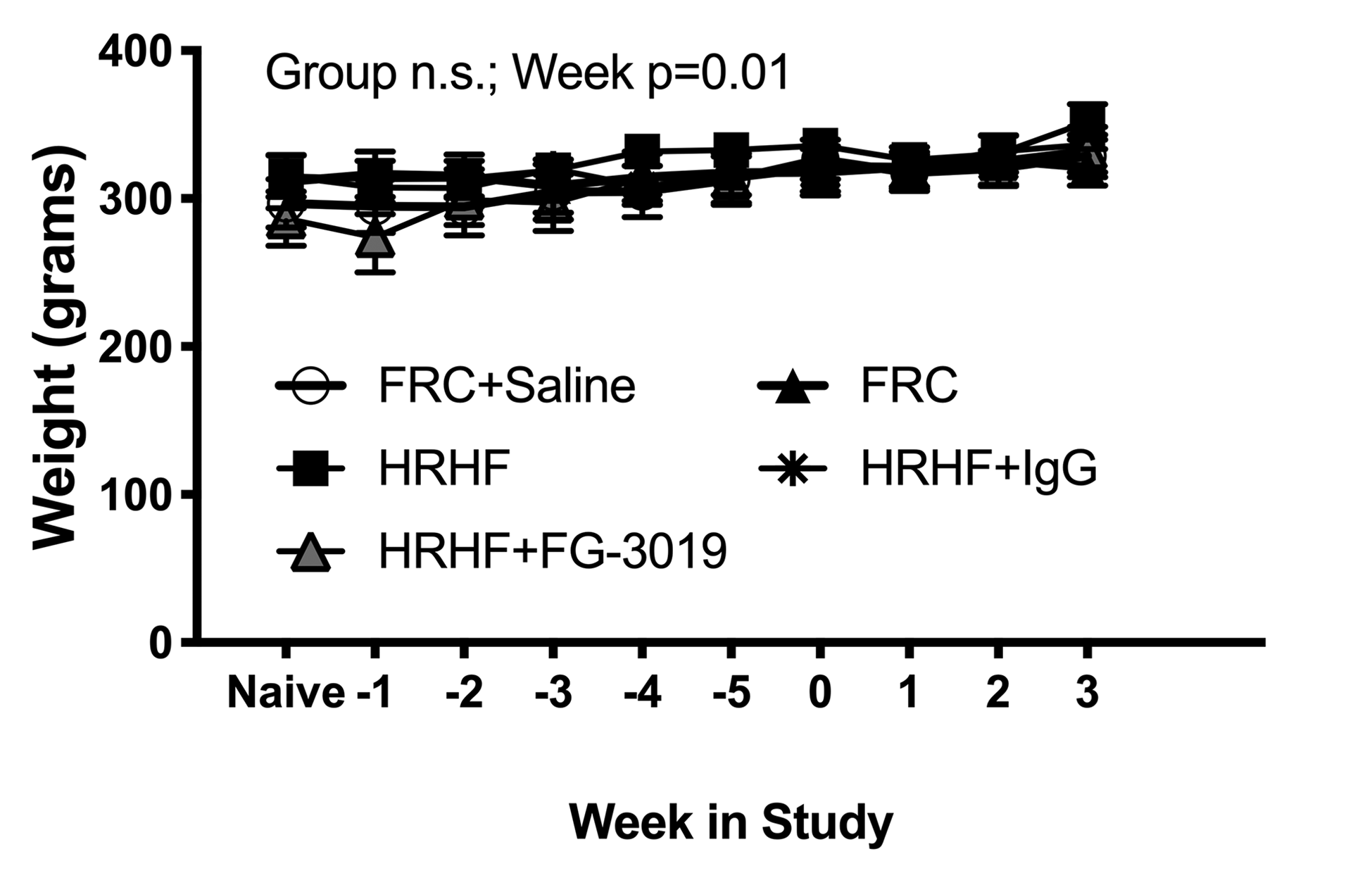

Supplement: Supplementary file 3 — Supporting information [file JOR-37-2004-s003.tif]

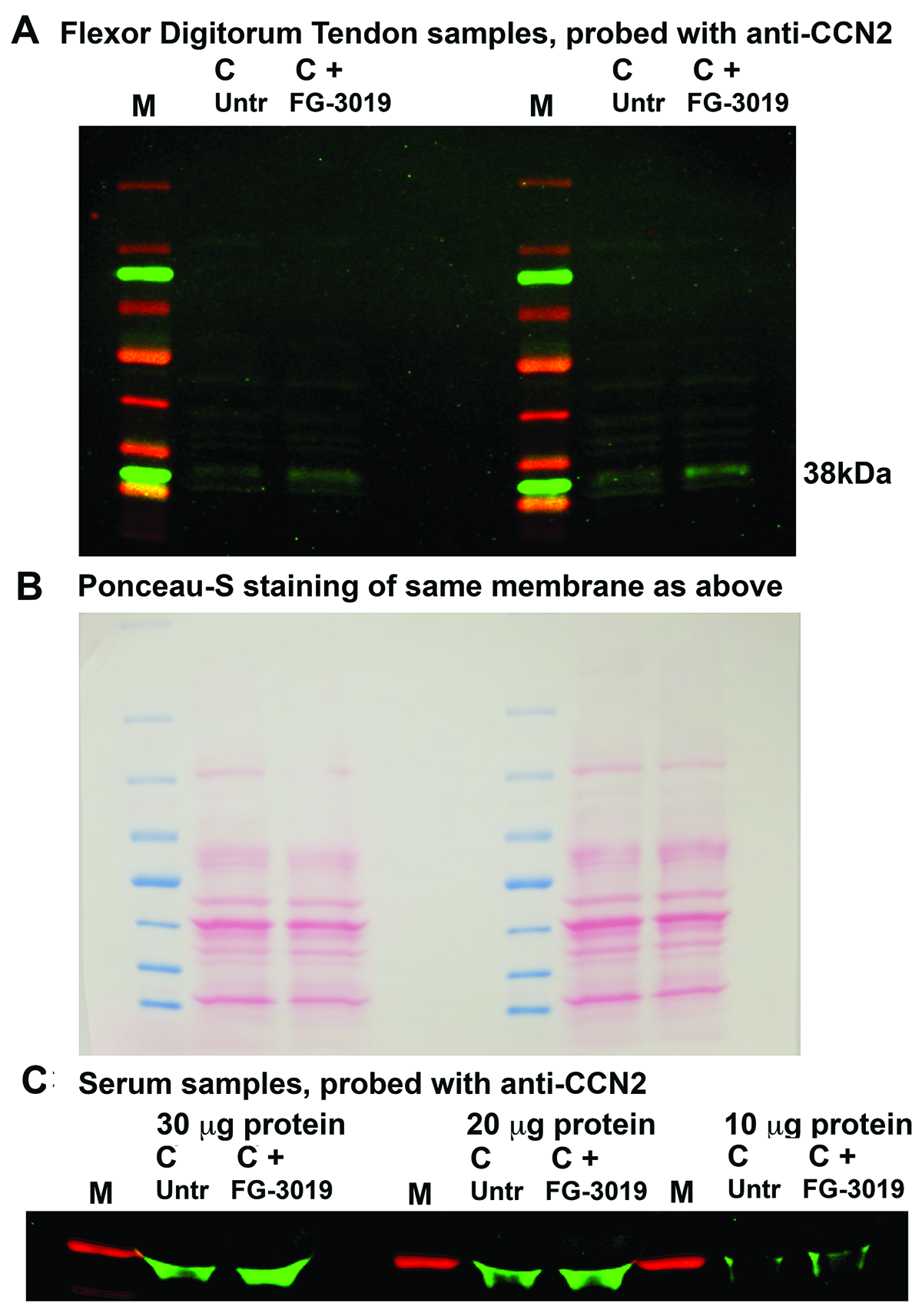

Supplement: Supplementary file 4 — Supporting information [file JOR-37-2004-s004.tif]

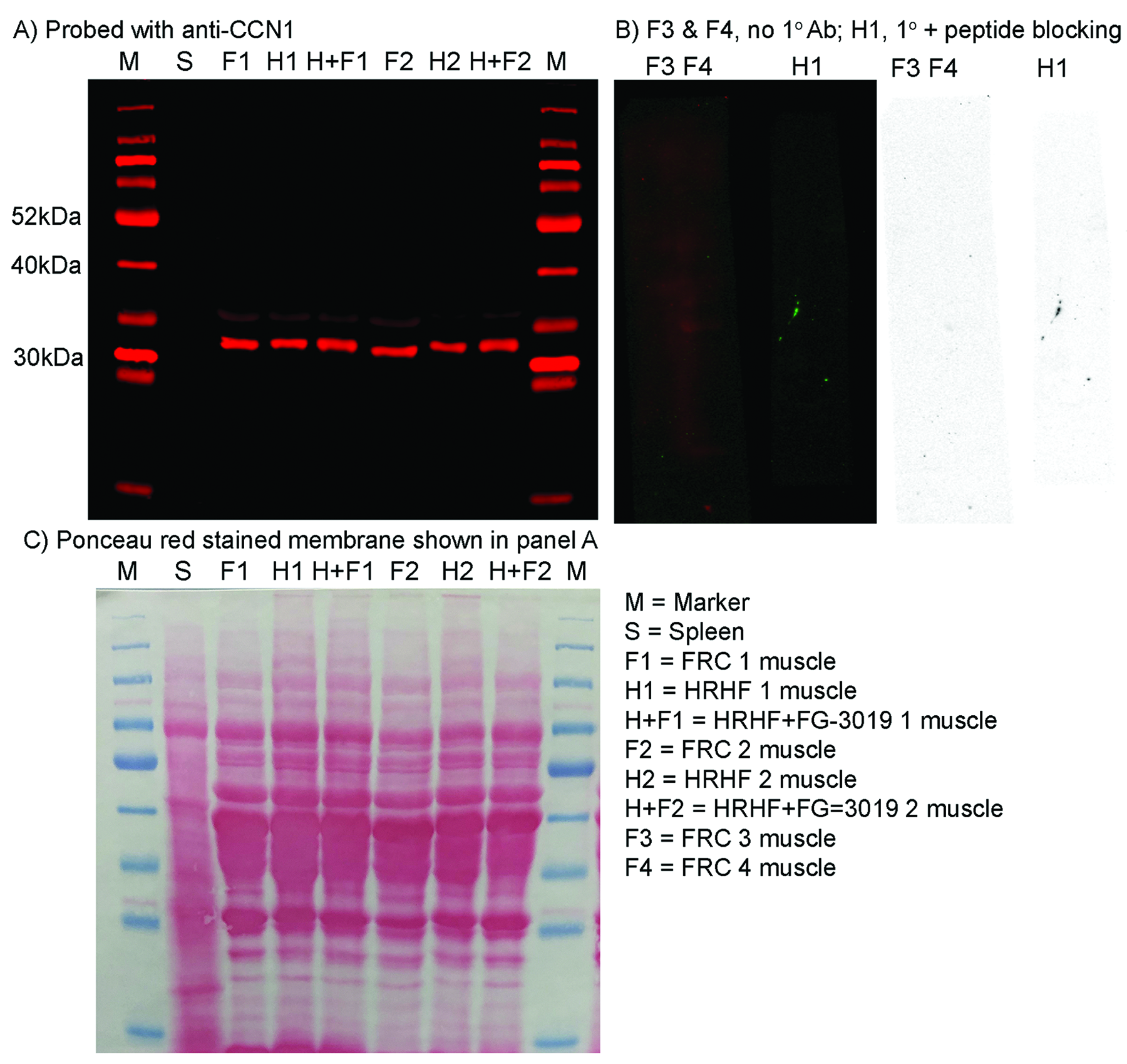

Supplement: Supplementary file 5 — Supporting information [file JOR-37-2004-s005.tif]

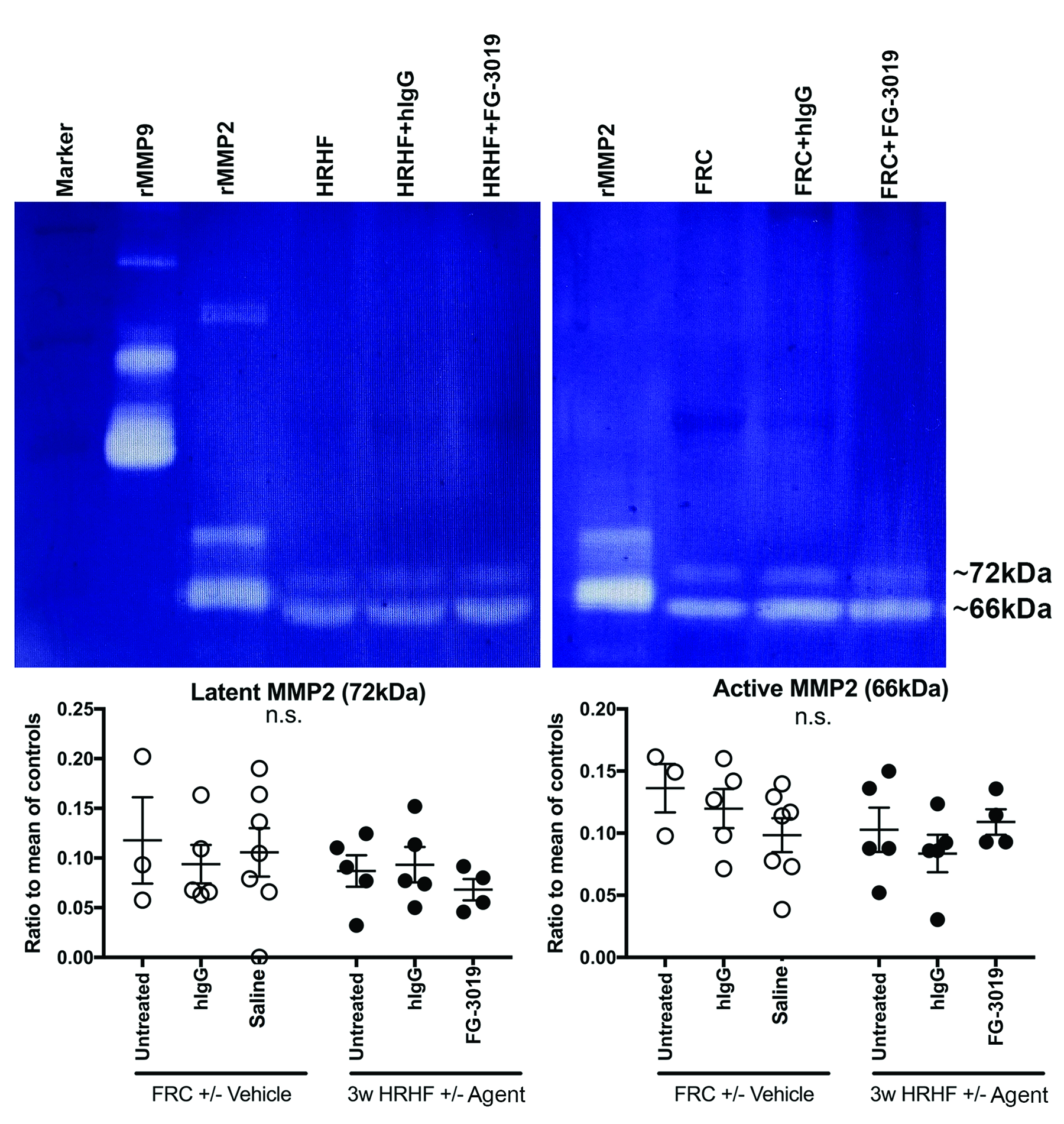

Supplement: Supplementary file 6 — Supporting information [file JOR-37-2004-s006.tif]

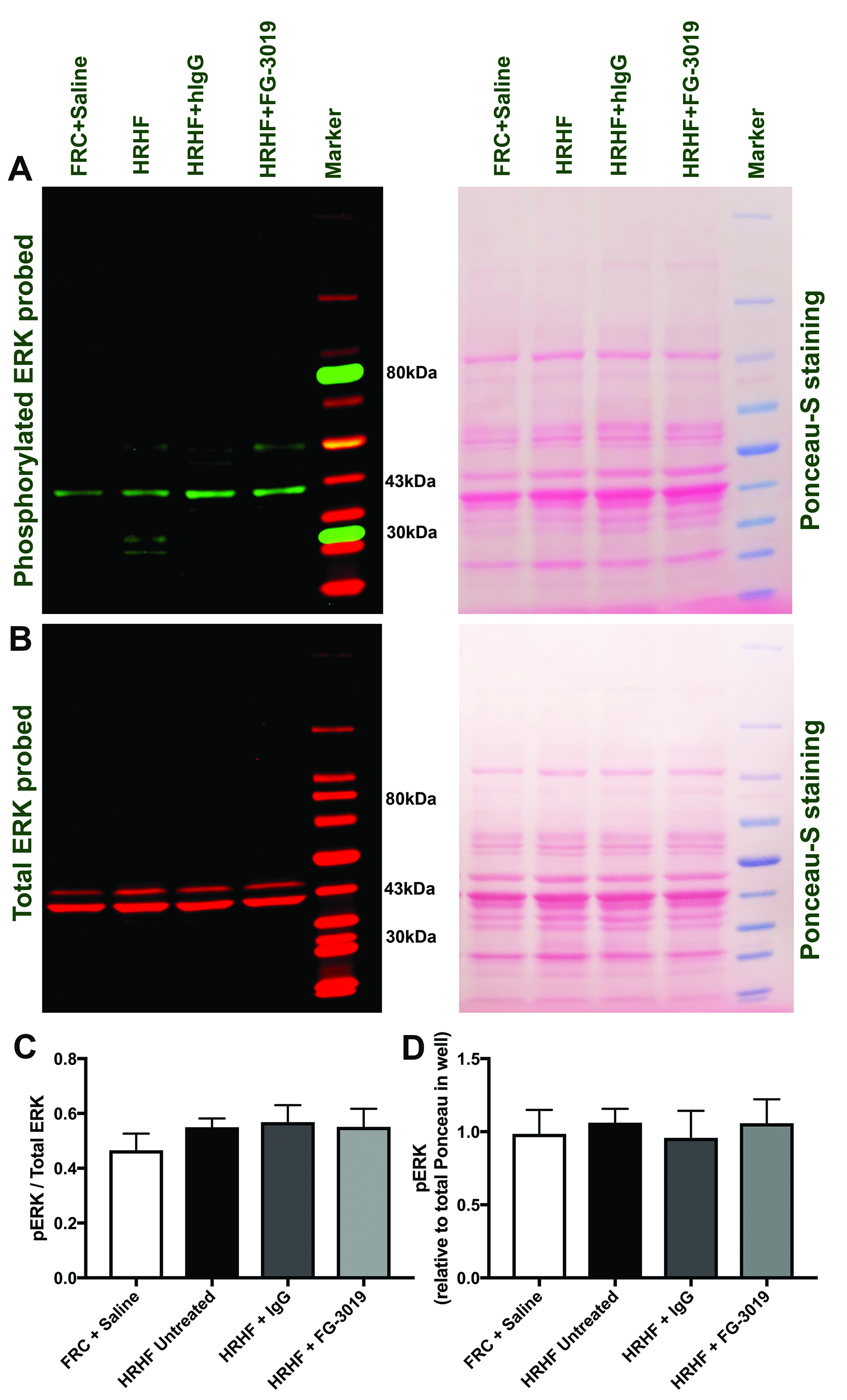

Supplement: Supplementary file 7 — Supporting information [file JOR-37-2004-s007.tif]
